# Effects of exposure to water disinfection by-products in a swimming pool: A metabolome-wide association study

**DOI:** 10.1016/j.envint.2017.11.017

**Published:** 2018-02

**Authors:** Karin van Veldhoven, Pekka Keski-Rahkonen, Dinesh K. Barupal, Cristina M. Villanueva, Laia Font-Ribera, Augustin Scalbert, Barbara Bodinier, Joan O. Grimalt, Christian Zwiener, Jelle Vlaanderen, Lützen Portengen, Roel Vermeulen, Paolo Vineis, Marc Chadeau-Hyam, Manolis Kogevinas

**Affiliations:** a MRC/PHE Centre for Environment and Health, Department of Epidemiology and Biostatistics, School of Public Health, Imperial College London, London, United Kingdom; b International Agency for Research on Cancer (IARC), Lyon, France; c ISGlobal, Centre for Research in Environmental Epidemiology (CREAL), Barcelona, Spain; d Universitat Pompeu Fabra (UPF), Barcelona, Spain; e CIBER Epidemiología y Salud Pública (CIBERESP), Barcelona, Spain; f IMIM (Hospital del Mar Medical Research Institute), Barcelona, Spain; g Department of Environmental Chemistry, Institute of Environmental Assessment and Water Research (IDÆA-CSIC), Barcelona, Spain; h Center for Applied Geoscience, Environmental Analytical Chemistry, University of Tuebingen, Tuebingen, Germany; i Institute for Risk Assessment Sciences (IRAS), Utrecht University, Utrecht, The Netherlands; j Italian Insitute for Genomic Medicine (IIGM), Turin, Italy

**Keywords:** Disinfection by-products, DBPs, Metabolome, LC-MS, Blood, Exposome

## Abstract

**Background:**

Exposure to disinfection by-products (DBPs) in drinking water and chlorinated swimming pools are associated with adverse health outcomes, but biological mechanisms remain poorly understood.

**Objectives:**

Evaluate short-term changes in metabolic profiles in response to DBP exposure while swimming in a chlorinated pool.

**Materials and methods:**

The PISCINA-II study (EXPOsOMICS project) includes 60 volunteers swimming 40 min in an indoor pool. Levels of most common DBPs were measured in water and in exhaled breath before and after swimming. Blood samples, collected before and 2 h after swimming, were used for metabolic profiling by liquid-chromatography coupled to high-resolution mass-spectrometry. Metabolome-wide association between DBP exposures and each metabolic feature was evaluated using multivariate normal (MVN) models. Sensitivity analyses and compound annotation were conducted.

**Results:**

Exposure levels of all DBPs in exhaled breath were higher after the experiment. A total of 6,471 metabolic features were detected and 293 features were associated with at least one DBP in exhaled breath following Bonferroni correction. A total of 333 metabolic features were associated to at least one DBP measured in water or urine. Uptake of DBPs and physical activity were strongly correlated and mutual adjustment reduced the number of statistically significant associations. From the 293 features, 20 could be identified corresponding to 13 metabolites including compounds in the tryptophan metabolism pathway.

**Conclusion:**

Our study identified numerous molecular changes following a swim in a chlorinated pool. While we could not explicitly evaluate which experiment-related factors induced these associations, molecular characterization highlighted metabolic features associated with exposure changes during swimming.

## Highlights

• MWAS of exposure to Disinfection By-Products from swimming in a chlorinated pool
• 6471 metabolic features were detected by UHPLC-QTOF- Mass Spectrometry.
• DBP exposures were measured in exhaled breath, urine and in swimming water.
• 293 metabolic features associated to at least one DPB, and 137 to all exposures
• Annotation revealed 13 compounds involved in the tryptophan metabolism pathway.

## 1 Introduction

Physical exercise, including swimming, is highly recommended because of its positive effects on general health. However, the health of swimmers might be at risk due to disinfection methods used in swimming pools, which lead to the formation of disinfection by-products (DBPs). DBPs are present in swimming pool water, and residential sources such as drinking water, shower and bath water. They result from the disinfectants (such as chlorine) reacting with natural organic matter (such as saliva, hair, or perspiration in the case of swimming pools). During swimming, showering and bathing, inhalation and dermal absorption are the main exposure routes, which lead to high concentrations of skin permeable and volatile DBPs in the blood, such as trihalomethanes (THMs).(Villanueva and Font-Ribera, 2012) Exposure from ingestion of water also occurs during swimming. More than 600 DBPs have been identified, including THMs and haloacetic acids (HAAs), and the International Agency for Research on Cancer (IARC) has classified several of them as possibly carcinogenic to humans (group 2B). (World Health Organization, 2004b)

Several epidemiological studies have investigated health effects of long-term exposure to residential DBPs and all identified an association with increased risk of bladder cancer. (Costet et al., 2011, Villanueva et al., 2004, Villanueva et al., 2015) Short-term effects of DBP exposure in swimming pools have been suggested to comprise increased lung epithelium permeability, in both adults(Font-Ribera et al., 2010) and children(Bernard et al., 2003). Asthma development and other respiratory complications have been related to long-term exposure in swimming pools.(Levesque et al., 2006, Villanueva et al., 2015) This has most consistently been observed among those who are occupationally exposed, such as swimming pool workers and professional swimmers.(Goodman and Hays, 2008, Thickett et al., 2002) In addition, some epidemiological studies have suggested potential adverse reproductive and developmental effects(Villanueva et al., 2015), but these findings were not validated in a recent large European study.(Kogevinas et al., 2016)

Several studies have suggested the genotoxic and mutagenic potential of some DBPs.(Beddowes et al., 2003, Du et al., 2013, Honer et al., 1980, Khallef et al., 2015, Kogevinas et al., 2010, Pals et al., 2013, Pals et al., 2011, Richardson et al., 2010, Stayner et al., 2014, Wang et al., 2014, Yuan et al., 2005) Specifically, higher levels of biomarkers of genotoxicity such as changes in micronuclei (MN) and DNA damage (comet assay) in peripheral blood lymphocytes have been reported in relation to brominated THM concentrations (excluding chloroform) in exhaled breath.(Kogevinas et al., 2010) Increased levels of markers of genotoxicity in maternal binucleated lymphocytes were also identified during the first and second trimester of pregnancy in relation to THM exposure from residential water (Khallef et al., 2015, Stayner et al., 2014) and swimming pool water(Honer et al., 1980, Richardson et al., 2010).

Experimental studies in cell lines (Beddowes et al., 2003, Yuan et al., 2005) and in blood biosamples (Du et al., 2013, Pals et al., 2013, Pals et al., 2011, Wang et al., 2014) have identified a link between genotoxicity/mutagenicity and oxidative stress, notably through the production of reactive oxygen species following exposure to various forms of DBPs.

The World Health Organization (WHO), the US Environmental Protection Agency (EPA) and other agencies have set drinking water guideline values or regulations for various THMs. (The National Primary Drinking Water Regulations, World Health Organization, 2011) In the US, levels of haloacetic acids (HAA) are also regulated, while this presently is not the case in Europe.(Union, 2015; United States Environmental Protection Agency) For swimming pool water, far fewer regulations are in place, although mutagenic levels in swimming pool water were found to be similar to those of drinking water.(Richardson et al., 2010)

The simultaneous acquisition of information of hundreds or thousands of metabolites in biospecimens from human subjects exposed to environmental toxicity has been used to successfully identify new biomarkers of exposure or effect, and to generate new hypotheses on possible mechanisms linking exposures to diseases (Bonvallot et al., 2013, Yuan et al., 2016, Zhang et al., 2016). In the present study, we adopt a metabolome-wide association study (MWAS) approach to identify possible changes in metabolic profiles after swimming in a chlorinated swimming pool. Our study was implemented in an experimental setup and features repeated measurements (before and after the swim) of both exposures and metabolic profiles for each participant. To accommodate this multiple measurement design, we used a flexible multivariate normal (MVN) model to regress levels of each metabolic feature against the measured exposure levels. This approach, coupled with an extensive effort to identify associated metabolic features, has the potential to help unravel molecular pathways affected by exposure to DBPs and ultimately inform on the mechanisms explaining the underlying toxicity. Therefore, the overall aim of our study is to investigate the short-term effects of DBP exposure on the metabolome and more specifically, the possible involvement of metabolic pathways linking DBP exposure and adverse health outcomes.

## 2 Materials and methods

### 2.1 Participants and samples

The participants for this study were part of the PISCINA II study, an experimental study performed in a 25 m long indoor chlorinated pool in Barcelona, Spain, between June and December 2013. It included 116 volunteers, aged 18–40 years, non-smoking and non-professional swimmers, who swam for 40 min at a leisurely pace in the swimming pool, resting at their own initiative. Four participants were evaluated per day, between 9 am and 2 pm (before having lunch).

From these 116 volunteers, 60 were selected for subsequent metabolomic profiling. Selection criteria were defined to ensure that: i) complete exposure measurements in the swimming pool were available, ii) biological samples were available; iii) there was an even proportion of men and women; iv) data on physical activity, lifestyle, and all possible adjustments covariates (see below) were available. For each of these 60 participants, blood samples collected by venepuncture using BD Vacutainer R Push Button Blood Collection Set with Pre-Attached Holder in a room detached from the swimming pool, in two occasions, before and 2 h after swimming. For this analysis, blood was collected in serum tubes. Samples were kept at 4 °C and were sent to the laboratory to be processed. After 1 h of free coagulation, samples were centrifuged at 4 °C and 0,25 mL of serum was stored in Screw Cap Micro Tubes at − 80 °C.

All participants were requested not to visit any swimming pools one week before the experiment and not to shower on the morning of the experiment. Information about water consumption and use, activities, transport, diet and medication was obtained from the participants through questionnaires before the start of the experiment. Height and weight were measured and body mass index (BMI) was calculated by dividing the weight in kg by the height squared in meters. During the experiment, swimming distance and swimming duration were measured. Energy expenditure (kcal) was estimated using the speed of swimming and the participant's weight, assuming 8.3 METs (metabolic equivalent tasks: kcal per kg per hour) of expenditure for swimming at 46 m/min according to the following equation based on work form Ainsworth et al. (2011):

Heart rate (beats/min) was recorded at every second using a Polar RCX5 heart rate monitor.

Informed consent was provided by each participant before commencement of the experiment. The study was approved by the ethics committee of the research centre according to national and international regulations.

### 2.2 Exposure variables

Two main types of exposures to DBPs were collected during the study: external exposure as measured by levels of DBPs in the swimming pool water, and internal exposures as estimated by measurements in exhaled breath for prioritised exposures and in urine for TCAA.

***External exposure*** consisted of DBPs measured in samples of swimming pool water collected in the pool, while the participants were swimming. Water samples were stored at 4 °C. As detailed elsewhere (Font-Ribera et al., 2016), chemicals measured included free chlorine, total organic carbon and total organic halogen, as well as 6 categories of DBP, each comprising various numbers of species: trihalomethanes (THM, 4 species), haloacetic acids (HAA, 9 species), haloacetonitriles (3 species), haloketones (1 species), nitrosamines/nitramines (2 species) and chloramines (3 species).

***Internal exposure*** consisted of DBPs measured in exhaled breath, including 4 trihalomethanes (chloroform (CHCl₃), bromodichlorormethane (BDCM), dibromochloromethane (DBCM), and bromoform (CHBr₃)). These measurements were obtained before swimmers entered the swimming pool and immediately after they exited the swimming pool, using the Bio-VOC™ Sampler (Markes International Ltd., UK). These chemicals were assessed by gas chromatography coupled to a mass spectrometer (GC–MS). Details on sample collection and analysis have been published previously.(Font-Ribera et al., 2016) All species were analysed separately and in combination, such as the total trihalomethanes (TTHM = Cl₃CH + BDCM + DBCM + Br₃CH) and the brominated trihalomethanes (BrTHMs = BDCM + DBCM + Br₃CH).

Trichloroacetic acid (TCAA) concentrations were measured for all participants in urine samples collected before and 30 min after swimming, using solid phase extraction followed by liquid chromatography tandem mass spectrometry (LC-MS/MS) as detailed previously.(Salas et al., 2014) Creatinine was measured in urine in order to adjust for dilution and TCAA concentration was expressed as creatinine adjusted levels (mmol TCAA/mol creatinine).

### 2.3 Metabolomic analyses

#### 2.3.1 Sample analysis

Samples were prepared by mixing 20 μL of serum with 100 μL of acetonitrile in polypropylene microcentrifuge tubes, centrifuging at 13000 RPM at 4 °C for 10 min and collecting 50 μL of the supernatant into a glass autosampler vial. After randomization into the batch, samples were analysed as a single uninterrupted batch with an ultra-high performance liquid chromatography-quadrupole time-of-flight mass spectrometry (UHPLC-QTOF-MS) system (Agilent Technologies, Santa Clara, USA) consisting of a 1290 Binary LC system, a Jet Stream electrospray ionization (ESI) source, and a 6550 QTOF mass spectrometer. Autosampler tray was kept refrigerated at 5 °C and 1 μL of the sample solution was injected on an ACQUITY UPLC HSS T3 column (2.1 × 100 mm, 1.8 μm; Waters). Column temperature was 45 °C and mobile phase flow rate 0.4 mL/min, consisting of ultrapure water and LC-MS grade methanol, both containing 0.1% (v/v) of formic acid. The gradient profile was as follows: 0–6 min: 5% → 100% methanol, 6–10.5 min: 100% methanol, 10.5–12.5 min: 5% methanol. The mass spectrometer was operated in positive polarity using following conditions: drying gas (nitrogen) temperature 175 °C and flow 12 L/min, sheath gas temperature 350 °C and flow 11 L/min, nebulizer pressure 45 psi, capillary voltage 3500 V, nozzle voltage 300 V, and fragmentor voltage 175 V. Data was acquired using extended dynamic range mode across a mass range of 50–1200, with a scan rate of 1.67 Hz. Continuous mass axis calibration was performed by monitoring two reference ions throughout the runs (*m*/*z* 121.050873 and *m*/*z* 922.009798). The analytical run was initiated with priming injections of a pooled quality control (QC) sample to achieve stable instrument response, followed by study samples that were intervened after every 10 injections with a QC sample and a solvent blank to monitor instrument performance.

#### 2.3.2 Data processing

Pre-processing of the acquired data was performed using Qualitative Analysis B.06.00 SP1, DA Reprocessor, and Mass Profiler Professional 12.1 software (Agilent Technologies, Santa Clara, CA, USA). The initial processing was performed using find by molecular feature (MFE) algorithm. Threshold values for mass and chromatographic peak heights were 500 and 5000 counts, respectively, with a compound quality score threshold at 70. Only singly charged ions were included. The resulting features were combined into a single list using 0.1 min retention time window for alignment, and those existing in at least 2% of all the samples were used as targets for a recursive feature extraction of the raw data using a find by formula (FBF) algorithm. For the recursive process, match tolerances were ± 30 ppm and ± 0.1 min for the mass and retention time, respectively. Ion species were limited to [M + H]^+^ and [M]^+^, without filtering by peak height or quality score. Resulting files were merged to generate a final data matrix, which was exported as a .csv file for statistical analyses.

### 2.4 Statistical analysis

Correlations between the exposures and measurements of physical activity were calculated using Spearman correlation coefficients. Exposures for which > 25% of the measurements were missing (*i.e.* across participants and for both sampling occasions, before and after the swim) were excluded from subsequent analyses.

.

Because the distribution of most metabolic features was skewed a log-transformation was performed on all features. Metabolic features that were detected in < 60% of the samples were excluded from the analysis and missing data in the remaining features were imputed using a quantile regression approach for left-censored missing data, implemented in the imputeLCMD R package (Lazar, 2015).

A common set of potential confounders at baseline was included in all statistical models: age, sex and BMI. In addition, the month at which the swimming experiment took place, pH of the water and temperature of the water and air (°C) were also considered as potential confounders.

Multivariate normal regression models were run to identify changes in metabolic features induced by the swim. MVN models accommodate repeated measure design upon setting the participant ID as grouping factor and assuming an unstructured variance covariance across observations per participants (before and after the swim).

As a benchmark model (Model 1), we regressed log transformed and standardised metabolite intensities (as outcome variable) against measured levels of each of the DBPs in exhaled breath, which were centred on the level before the swim. To disentangle the effect of physical activity from that of exposures themselves, we also ran Model 1 additionally adjusting for physical activity as measured by energy expenditure (Model 2). As an alternative to Model 1, we ran our metabolome-wide MVN model using the binary pre-post swimming indicator as a proxy for the experimentally-induced exposure changes (Model 1’). As a sensitivity analysis and in order to identify potential effect of exposure(s) that would not be captured by the pre-post swimming indicator, but by exposures only, we further adjusted Model 1 for this binary variable.

We ran the same set of models in relation to exposures measured in urine (TCCA), and in the swimming pool water (DBPs). For the latter, only one measurement was available during the swim. Models were parametrised setting the exposure before the swim to 0 and to the measured value after the swim. To investigate the consistency of the metabolic features found associated to internal exposures (measured in exhaled breath and urine) and those associated to external exposures (measured in swimming water), we ran a principal component analysis on both sets of metabolic features (using measurements after the swim) and evaluated the pairwise correlation across the two sets of principal components.

In all models, associations were declared significant based on a Bonferroni corrected significance level (ensuring a family-wise error rate < 0.05).

For models on exhaled breath exposures, we also performed series of stability analyses assessing the robustness of our findings to outlying observations. Specifically, we ran the models on 1000 random subsamples (90% of the study population), and reported the number of times each of the associations we identified in the main analysis was declared significant across the 1000 sub-populations.

All analyses were performed using R version 3.1.3 (2015-03-09).

### 2.5 Annotation of metabolic features

Annotation of the discriminating features was done in four steps: 1) The *m*/*z* values of all the features were searched against the human metabolite database (HMDB, www.hmdb.ca, as of 25th May 2016) using [M + H]^+^ and [M + Na]^+^ as adducts and ± 8 ppm for molecular weight tolerance, with the metabolite origin set to Toxin/Pollutant, Exogenous, and Endogenous. 2) Features with HMDB hits were grouped based on retention time similarity and intensity correlation across the samples to assist in identifying ions originating from the same metabolite. 3) Quality of the chromatographic peaks and spectra were inspected and the plausibility of HMDB candidates was assessed based on retention time, isotope pattern and adduct formation. 4) Identification was confirmed by reanalysis of representative samples and pure standards when available and comparison of the retention times and the MS/MS spectra acquired at 10 V, 20 V, and 40 V collision energies. When standards were not available, MS/MS spectra were acquired and compared against those in mzCloud (www.mzcloud.org) or Metlin (metlin.scripps.edu). The level of identification was based on the recommendations of the Chemical Analysis Working Group of Metabolomics Standards Initiative.(Sumner et al., 2007) In our discussion we mainly refer to Level 1 (highest).

For the features that could not be identified, the presence of chlorine atoms was studied. A compound exchange file (.cef) was generated for all the discriminant features in Mass Profiler Professional and exported to MassHunter Qual software for formula generation. The presence of 1–3 Cl atoms were required in addition to C (*n* = 2–50), H (*n* = 1–100), O (*n* = 0–10), N (*n* = 0–10), and S (*n* = 0–1), with maximum allowed mass error of 5 ppm and minimum score value of 50. Isotope patterns of the features fulfilling these conditions were inspected, and those with isotopic mass defects between the M and M + 1 peaks within + 0.0015 and + 0.0045, and between M and M + 2 peaks within − 0.0015 and − 0.0045 were considered chlorinated compounds. This corresponds to a ± 3-ppm allowable deviation from the theoretical values for a chlorinated compound with an *m*/*z* of 500. The principle has been described in detail elsewhere (Thurman and Ferrer, 2010).

## 3 Results

### 3.1 Study population and DBP exposures

The characteristics of the study population and of the swimming pool environment are reported in Table 1. Study participants were 50% male and 50% female, aged between 18 and 37 years, with an average BMI of 23.7 kg/m^2^. Most swims took place in September, and the average swimming distance was 996.2 m, the average swimming time, 31.8 min, the average %HR_max69_, 62.6, and average energy expenditure, 204.2 kcal.

**Table 1:** Characteristics of study population (*n* = 60) and swimming pool environment

Study population	Number (%)^a^		
Sex			
Male	30 (50)		
Female	30 (50)		

Month of experiment			
June	13 (21.7)		
September	21 (35.0)		
October	10 (16.7)		
November	8 (13.3)		
December	8 (13.3)		


	Mean	SD	Range
Age (years)	25.04	5.25	18–37
Height (cm)	169.0	8.37	152.7–186.0
Weight (kg)	68.22	13.02	43.90–107.50
BMI	23.74	3.29	16.55–32.46

Physical activity			
Swimming distance (m)	996.2	317.2	200.0–1850.0
Swimming time (min)	31.84	8.00	7.00–42.00
%HR_max69_^b^	62.62	29.50	0.00–99.09
Energy expenditure (kcal)	204.2	73.64	58.34–417.30

Swimming pool environment			
pH	7.51	0.21	7.1–7.9
Water temperature (°C)	27.97	0.30	27.2–28.6
Air temperature (°C)	28.19	0.73	26.5–29.5

a Percentages do not always add to 100% due to missing values.

b %HR_max69_ percentage of HR values > 69% HR_max_ (indicates high intensity physical activity).

Measurements of Dimethylnitrosamine (DMNA) and Nitrosodimethylamine (NDMA) in the swimming water were missing for 68 and 57% of the study population, respectively, and were excluded form subsequent analyses.

**Table 2:** Description of exposures measured in exhaled breath and in swimming pool water.

Exhaled breath	N	Before swimming	After swimming	Paired *t*-test
		Mean ± SD (range)	Mean ± SD (range)	*P*-value
Trihalomethanes (μg/m^3^)				
Chloroform (CHCl₃)	60	0.44 ± 0.31 (0.09–1.51)	11.54 ± 4.74 (2.69–26.31)	< 2.2e–16
Bromodichloromethane (BDCM)	60	0.06 ± 0.05 (0.00–0.23)	2.49 ± 1.19 (0.38–6.43)	< 2.2e–16
Dibromochloromethane (DBCM)	60	0.02 ± 0.03 (0.00–0.12)	0.54 ± 0.32 (0.08–1.75)	< 2.2e–16
Bromoform (CHBr₃)	60	0.03 ± 0.02 (0.00–0.14)	0.11 ± 0.07 (0.02–0.40)	1.875e–12
Brominated THMs (BrTHM)	60	0.11 ± 0.10 (0.02–0.45)	3.14 ± 1.57 (0.48–8.59)	< 2.2e–16
Total THMs (TTHM)	60	0.55 ± 0.37 (0.11–1.67)	14.68 ± 6.06 (3.33–32.57)	< 2.2e–16


Swimming pool water	N	Mean ± SD	Range
Trihalomethanes (μg/L)			
Chloroform (CHCl₃)	53	37.53 ± 8.16	25.39–60.58
Bromodichloromethane (BDCM)	53	7.78 ± 2.31	3.83–12.90
Dibromochloromethane (DBCM)	53	2.65 ± 1.03	1.14–4.70
Bromoform (CHBr₃)	53	1.01 ± 0.53	0.23–1.91
Brominated THMs (BrTHM)	53	11.43 ± 3.70	5.77–19.36
Total THMs (TTHM)	53	48.96 ± 9.84	31.16–73.56
Dichloroacetic acid (DClAA)	60	29.47 ± 10.30	15.40–51.65
Trichloroacetic acid (TClAA)	60	59.90 ± 10.48	39.20–83.40
Bromochloroacetic acid (BrClAA)	60	5.23 ± 1.58	2.40–8.80
Dibromoacetic acide (DBrAA)	60	1.50 ± 0.73	0.50–3.10
Dibromochloroacetic acid (DClBrAA)	60	12.70 ± 5.22	4.80–23.36
Total haloacetic acids (THAA)	60	109.10 ± 20.94	73.30–144.30

Haloacetonitriles (μg/L)			
Dichloroacetonitrile (C₂HCl₂N)	53	7.16 ± 1.86	4.09–11.62
Bromochloroacetonitrile (CHBrClCN)	49	3.63 ± 0.81	1.84–4.70
Total organic carbon (mg/L) (NPOC)	59	2.77 ± 1.24	1.78–10.11
Free chlorine (mg/L) (FCl)	59	1.33 ± 1.24	0.62–2.01
Haloketone (μg/L) (C₃H₃Cl₃O)	53	2.27 ± 0.78	1.39–4.67
Monochloramine (NH₂Cl)	59	0.31 ± 0.23	0.00–0.73
Dichloramine (NHCl₂)	58	0.24 ± 0.23	0.00–0.63
Trichloramine (NCl₃)	59	0.11 ± 0.23	0.00–1.64


Urine	N	Before swimming	After swimming	Paired *t*-test
		Mean ± SD (range)	Mean ± SD (range)	*P*-value
Creatinine adjusted trichloroacetic acid TCAA (mmol TCAA/mol creatinine)	58	3.66 ± 3.41 (0.47–15.90)	10.05 ± 10.10 (0.91–44.44)	8.74e-07

We observed very high and consistent correlation levels across exposure concentrations measured in exhaled breath both before and after the experiment (Fig. S1), though these were strengthened after the swim (Fig. S1-B). Exposure to all four trihalomethanes measured in exhaled breath, as well as the total trihalomethanes and the brominated trihalomethanes were all significantly higher after the swimming experiment compared to levels measured before the swim (Table 2). In swimming pool water, the highest concentrations of trihalomethanes, haloacetic acids, haloacetonitriles, and chloramines were measured for chloroform (mean: 37.53 μg/L), dichloroacetic acid (mean: 29.47 μg/L) and trichloroacetic acid (mean: 59.9 μg/L), dichloroacetonitrile (mean: 7.16 μg/L), and monochloramines (mean: 0.31 mg/L), respectively.

Correlation across internal exposure measurements and external exposure measurements are summarised in Fig. S2. This plot suggests strong block correlation within internal exposure levels, and moderate correlations within exposures measurement in the water and between both types of exposure measurements.

### 3.2 Metabolic profiles

After imputation, a total of 6471 metabolic features were included in the analyses and observations from two participants were excluded due to missing values for some of the confounding factors. Analysis of QC samples indicated good reproducibility along the run with coefficients of variation < 5% for 10 model compounds: Caffeine, Glutamine, Glycocholic acid, Hippuric acid, Indole-3-acetic acid, Lauroylcarnitine, Methionine, Phenylalanine, Tyrosine, and Uric acid.

The number of metabolic features whose level was found significantly associated with DBP exposure levels measured in exhaled breath are shown in Table 3. In Model 1, we identified many metabolic features whose blood levels were associated with DBP exposures in exhaled breath: the number of associations ranged from 151 for CHBr_3_ to 269 for BDCM. These results were not affected by the additional adjustment on air temperature and water pH (results not shown), which were therefore not considered as possible confounders in subsequent analyses.

**Table 3:** Number of mass spectrometry features significantly associated with each THM level measured in exhaled breath after Bonferroni correction for multiple testing.

THM levels in Exhaled breath (μg/m^3^)	Model 1^a^	Model 2^b^
Chloroform (CHCl₃)	249	4
Bromodichloromethane (BDCM)	269	1
Dibromochloromethane (DBCM)	222	0
Bromoform (CHBr₃)	151	0
Brominated THMs (BrTHM)	258	1
Total THMs (TTHM)	258	1

a Model 1 is adjusted for age, sex, BMI as confounders.

b Model 2 is adjusted for age, sex, BMI, energy expenditure (kcal).

**Fig. 1:**
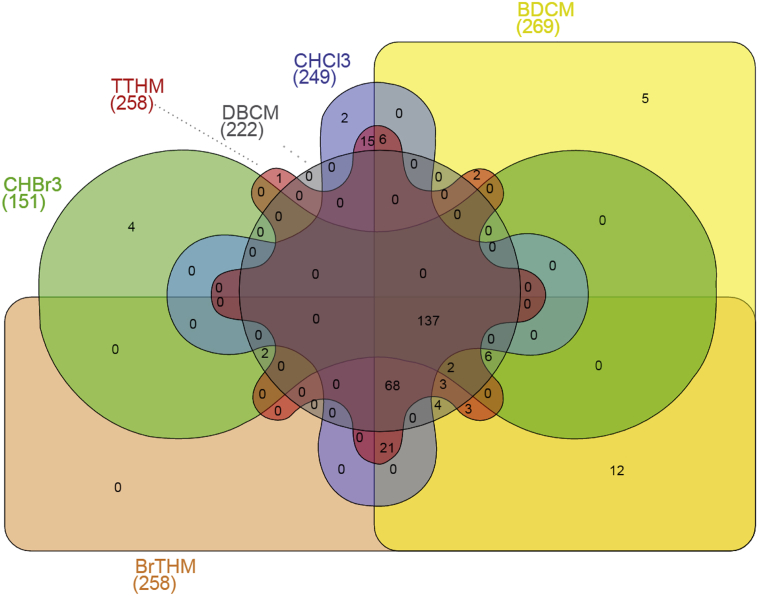
Venn diagram for the 293 metabolic features found associated with at least one exposure measured in exhaled breath using Model 1.

Our model identified a total of 293 metabolic features that were associated with at least one exposure level. Of these, 137 were found associated with all exposures (Fig. 1), and only 12 were found exclusively associated with a single exposure or a combination thereof: 5 for BDCM, 4 for CHBr_3_, 2 for CHCl_3_, while an additional compound was associated with TTHM (Table S1); all the metabolic features associated with DBCM, BrTHM, were found associated with at least one other exposure. Overall, of the 293 metabolic features (Table S1), aside from the 12 exclusive associations and of the 137 features associated with all exposures, we identified 29 features associated with two exposure, 15 with three exposures, 30 with four exposures, 70 with five exposures.

**Fig. 2:**
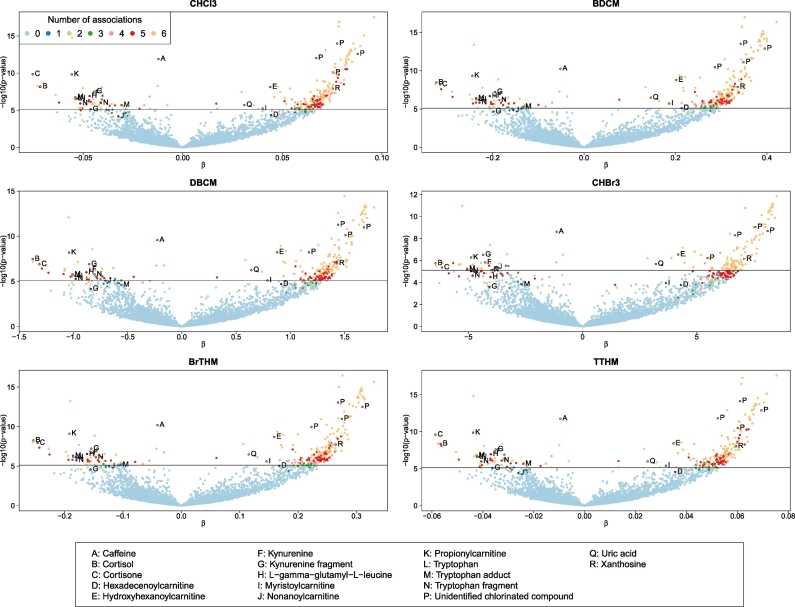
Results from the metabolome-wide association study for each exposure to THM measured in exhaled breath, using Model 1. Each dot represents a metabolic feature, and is represented by the –log_10_(*p*-value) measuring the strength of its association with THM levels as a function of the effect size estimates. Metabolic features are coloured according to the number of Bonferroni significant associations they exhibit across all six exposures. Light blue dots (*N* = 6178) are the metabolic features not found associated with any exposure. (For interpretation of the references to colour in this figure legend, the reader is referred to the web version of this article.)

The sign of the associations for all metabolic features was consistent across all six exposures (four individual THMs, brominated THMs and total THMs). Of the 293 metabolic features found associated to at least one exposure, 224 (76%) were upregulated with increased exposure levels (Fig. 2, Table S1). Of the exposure-associated metabolic features, a large proportion exhibited low *p*-values (from 21 to 48 associations with p-values below 10^− 10^ across all exposures). The strongest associations were those found associated with all exposures. In addition, and at least partially due to the strong exposure contrast before and after the experiment, none of the associations identified in Model 1 remained statistically significant while adjusting for the pre-post swimming binary indicator.

Stability analyses randomly subsampling 90% of the full study population revealed that between 82 and 84% of the metabolic features found associated with each exposure were also reported statistically significant in > 80% of the subsamples. This indicated that the associations we identified were not driven by outlying observations.

Visualisation of the pairwise correlation levels across the 293 exposure-related metabolic features (Fig. S3) showed strong clustering. This high level of correlation was further supported by principal component analyses, which showed that 12 and 26 components are necessary to explain 80 and 90% of the total variance of the 293 metabolic features.

In an alternative model using the pre-post binary indicator as a proxy for all exposures rather than measured exposure themselves, results were highly consistent with those from Model 1 that used exhaled breath THM levels: we identified 280 associated features at a Bonferroni-corrected significance level. Of these, 230 were among the 293 features (Fig. S4) found associated with at least one exposure level measured in exhaled breath, and of the 50 remaining metabolic features associated with the pre-post swimming binary indicator, > 30 had a *p*-value within one order of magnitude of Bonferroni corrected significance level (*p*-value < 5 × 10^− 5^).

Adjusting Model 1 for energy expenditure (kcal, Model 2) drastically reduced the number of statistically significant associations: 4 metabolic features were found associated with CHCl_3_, and one with BDCM, BrTHM and TTHM (Table 3). Additionally, the MWAS on energy expenditure identified 188 metabolic features whose p-value reached Bonferroni significance level. Of these, 161 were also found associated with at least one DBP exposure measured in exhaled breath. Overall, this suggests that the binary pre-post swimming indicator captures more than changes in DBP exposure induced by the swim, including physical activity and other experiment-related factors.

**Fig. 3:**
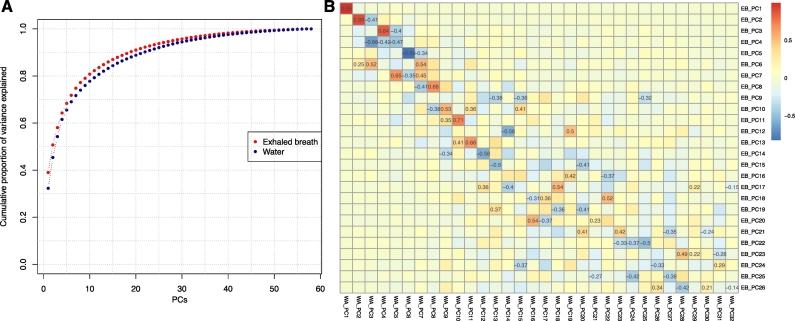
Comparison of the metabolic features identified using Model 1. The 333 and 293 metabolic features identified in the analyses of exposure measurement in water, and exhaled breath, respectively are summarised using Principal Component Analysis. Metabolic scores were calculated using measurements after the swimming experiment. We report in A the cumulative proportion of variance explained for the 293 features associated to exhaled breath exposures (in red) and the 333 associated to water exposures (in blue). We report in B the pairwise correlation for the scores obtained across the 32 and 26 PCs required to explain over 90% of the variance of the metabolic features associated with exposure levels measured in water/urine (X-axis) and exhaled breath (Y axis), respectively. (For interpretation of the references to colour in this figure legend, the reader is referred to the web version of this article.)

We ran Models 1 and 2 in relation to levels of trichloroacetic acid measured in urine, and DBP concentrations measured in swimming pool water, including THMs, haloacetic acids, haloacetonitriles, total organic carbon, free chlorine, haloketone and chloramines, as summarised in Table 4. As for the exposures measured in exhaled breath, several associations were found by Model 1, most of which did not reach statistical significance upon adjustment for energy expenditure (Model 2 in Table 4). All of the associations identified in Model 1 for water and urine exposure measurements lost statistical significance when adjusting on the pre-post experiment indicator. A total of 333 metabolic features were found associated with at least one of the 20 exposure levels. Of these, 225 were also found in the models for exposures measured in exhaled breath. To summarise the overlapping information between the 293 metabolic features identified in exhaled breath and the 333 features found for urine and water measurements, we ran principal component analysis of both subsets of metabolic features and investigated the pairwise correlation between each of the 26 and 32 components that were necessary to explain > 90% of the variance in the 293, and 333 metabolic features respectively (Fig. 3). Each of the first 6 PCs of the 293 metabolic features associated to a least one exposure level measured in exhaled breath, explaining 71% of the total variance (Fig. 3-A), are strongly correlated (Pearson's correlation coefficients > 0.65, in absolute value, Fig. 3-B) to at least one of the 6 first PC obtained from the analyses on water measurements (explaining 69% of the total variance; Fig. 3-A). This further supports the high level of consistency of the results found using water measurement of exposures.

**Table 4:** Number of metabolic features found associated with exposures measured in swimming pool water or urine samples.

Swimming pool water	Model 1^a^	Model 2^b^
Trihalomethanes (μg/L)		
Chloroform (CHCl₃)	168	1
Bromodichloromethane (BDCM)	166	0
Dibromochloromethane (DBCM)	146	0
Bromoform (CHBr₃)	133	0
Brominated THMs (BrTHM)	166	0
Total THMs (TTHM)	176	0

Haloacetic acids (μg/L)		
Dichloroacetic acid (DClAA)	163	1
Trichloroacetic acid (TClAA)	229	7
Bromochloroacetic acid (BrClAA)	182	2
Dibromoacetic acide (DBrAA)	128	2
Dibromochloroacetic acid (DClBrAA)	166	2
Total haloacetic acids (THAA)	214	2

Haloacetonitriles (μg/L)		
Dichloroacetonitrile (C_2_HCl_2_N)	173	11
Bromochloroacetonitrile (CHBrClCN)	232	0
Total organic carbon (mg/L) (NPOC)	106	1
Free chlorine (mg/L) (FCl)	249	0
Haloketone (μg/L) (C_3_H_3_Cl_3_O)	139	15

Chloramines (mg/L)		
Monochloramine (NH_2_Cl)	53	0
Dichloramine (NHCl_2_)	75	0
Trichloramine (NCl_3_)	0	0

Urine		
Creatinine adjusted trichloroacetic acid TCAA (mmol TCAA/mol creatinine)	18	0

a Model 1 includes age, sex, BMI as confounders.

b Model 2 includes age, sex, BMI, kcal as confounders.

**Table 5:** Identified metabolites from the list of 293 metabolic features found associated with at least one DBP exposure measured in exhaled breath.

Identity	*m*/*z*^a^	Retention time	*m*/*z* difference	Identification level^b^	Direction	Associated exposures
Uric acid	169.03598	1.150	0.0004	1	UP	DBCM, CHBr_3_
Propionylcarnitine	218.13858	1.361	− 0.0001	1	DOWN	DBCM
Kynurenine	209.09238	1.931	0.0003	1	DOWN	CHCl_3_, DBCM, CHBr_3_
Xanthosine	285.08318	1.980	0.0002	1	UP	BDCM, BrTHM, TTHM
Tryptophan	205.09798	2.599	0.0008	1	DOWN	All except CHBr_3_, TTHM
Caffeine	195.08818	3.300	0.0006	1	DOWN	All
Cortisone	361.20118	5.104	0.0003	1	DOWN	All except BDCM, BrTHM
Cortisol	363.21648	5.263	0.0001	1	DOWN	All except TTHM
Myristoylcarnitine	372.31098	6.039	0.0002	1	DOWN	CHCl_3_, BDCM, BrTHM
l-Gamma-glutamyl-l-leucine	261.14378	3.041	0.0007	2	DOWN	All except BDCM, CHBr_3_
Hydroxyhexanoylcarnitine	276.17968	2.490	0.0009	2	UP	All except DBCM
Nonanoylcarnitine	302.23228	4.716	0.0003	2	DOWN	CHBr_3_
Hexadecenoylcarnitine	398.32578	6.137	− 0.0007	2	UP	BDCM

Level 1 (identity confirmed): retention time and MS/MS matched with an authentic chemical standard; Level 2 (putative annotation): no standard available or analysed but mass within 5 ppm mass error and MS/MS spectra matches with those in a database.

a In case multiple ion species were detected, the most intense ion is represented

b Identification level indicates the degree of confidence in annotation (from reference 61).

Annotation was carried out for the 293 features found associated with a least one exposure measured in exhaled breath. Of these, 185 did not match by mass to any of the compounds in HMDB and were therefore excluded. The remaining 108 features were grouped by their retention time and intensity correlation to assist in finding ions representing the same metabolite. The resulting 49 feature groups were visually inspected for the quality of chromatographic peaks and mass spectra, and the plausibility of the HMDB candidates was assessed. Finally, 20 features could be identified corresponding to 13 different metabolites (see Table 5 and Fig. S5). A significant enrichment for the tryptophan metabolism pathway was observed following the swimming experiment identifying two metabolites, kynurenine and tryptophan that were down-regulated. Increased levels of xanthosine were associated to BDCM and total brominated THM and total THM, while lower levels of cortisol and cortisone were found to be associated with swimming.

Screening of all the 293 features for chlorine resulted in 75 features with isotope patterns suggesting the presence of 1–3 chlorine atoms. After grouping the features by retention time and applying the acceptance criteria for the isotopic mass defects, 4 individual compounds were found with strong evidence for the presence of chlorine in their elemental composition (Compounds 1–4, Fig. S6). Compounds 2–4 were detected as both [M + H]^+^ and [M + Na]^+^ ions, with the latter 11–19 times greater in intensity. MS/MS analysis of Compound 1 was unsuccessful, but other compounds yielded comparable product ion spectra with *m*/*z* 57.033 and 99.081 as the most intense common fragments. Fragmentation of the [M + Na]^+^ ions were expectedly less intense, but revealed a neutral loss of HCl, confirming the presence of Cl in the Compounds 2–4. Reanalysis of representative samples using negative ionization mode did not result in the detection of any of the four compounds, indicating a lack of anionic functionality such as carboxylic acid groups. The best-fitting computed formulas were C_13_H_11_ClN_2_O3_S_, C_15_H_31_ClO_8_, C_18_H_37_ClO_10_, and C_18_H_36_Cl_2_O_9_ for the Compounds 1–4, respectively. Searching these formulas in the chemical structure databases PubChem, TOXNET, and ChemSpider returned matches only for the Compound 1 (1679 results, ChemSpider), but the lack of MS/MS spectra for Compound 1 prevented in-silico fragmentation based screening of the database candidates for its structural elucidation.

## 4 Discussion

This paper describes the first study to investigate the effect on the metabolome of short-term exposure to DBPs during a swimming pool experiment. We identified numerous statistically significant associations between 40 min of swimming in a chlorinated swimming pool and changes in levels of metabolites in blood. Overall, models using exposure measurements and those using the binary pre-post swimming indicator provided highly consistent results. This suggests that most of the effective and co-occurring experimentally-induced factors affecting the metabolome are captured by the binary experimental indicator. However, for several exposures, in particular Bromoform in exhaled breath, and exposures measured in water and urine, the model using exposure levels found less associations than the model with the pre-post experiment. This may be attributed to a greater variability in these exposure measurements, which in turn can relate to differential measurement precision, or to additional confounding factors which do not relate to the swimming experiment.

There was a high correlation between physical activity, measured by kcal, and THM concentrations, notably due to the fact that the internal dose increases significantly with intensity of physical activity (Marco et al., 2015).

As illustrated in the analyses adjusting DBP exposures for physical activity, it is challenging to statistically disentangle the effect of exposures from that of physical activity (or of any other factor related to the swimming experiment) due to their strong correlation. However, through metabolite identification in combination with evidence from the literature related to DBP exposure we were able to link some of our associations to specific DBPs. More specifically, we used the annotated metabolic features associated with DBP levels to seek for metabolic pathways that would be perturbed by the swimming experiment. Analyses suggested a significant enrichment for the tryptophan metabolism pathway, in which we identified two metabolites, kynurenine and tryptophan that were down-regulated following the swimming experiment. Perturbation of this pathway might reflect a generic mechanism linked to physical overload, including kynurenine linked to oxidative stress and immunity (Moffett and Namboodiri, 2003). A study by Zhang et al. assessed perturbations of urine metabolites in mice induced by trichloroacetamide (a nitrogenous DBP), and also observed changes in the levels of tryptophan (Zhang et al., 2015), while another study exposed mice to monohaloacetamides and evaluated metabolic changes in comparison with non-exposed mice and found altered levels of kynurenine (Deng et al., 2014). Walker et al. investigated occupational exposure to trichloroethylene (TCE) in relation to untargeted plasma metabolomics, and similarly observed lower levels of tryptophan with increasing levels of TCE (Walker et al., 2016). Tryptophan metabolism plays a role in the generation of various neuroactive compounds within the central nervous system (CNS) (Lovelace et al., 2017) and exposure to (high) concentrations of certain DBPs has been shown to lead to depression of the CNS in both animal and human studies (U.S. Department of Health And Human Services, P.H.S, 2005, World Health Organization, 2004a). Moreover, a study of urinary metabolites of bladder cancer cases compared with non-bladder cancer controls found significant perturbations of the kynurenine pathway, with tryptophan levels consistently lower in cases (Pasikanti et al., 2013). Our results identifying exposure to DBP as affecting the tryptophan metabolism may therefore serve as an interesting hypothesis to investigate the contribution of these exposures in the risk of bladder cancer. However, some studies have also observed altered tryptophan and kynurenine levels after physical exercise (Daskalaki et al., 2015, Mukherjee et al., 2014, Pechlivanis et al., 2010, Xiao et al., 2016).

Among the annotated metabolites, caffeine was found to be inversely associated with all DBP levels. This result may indicate that we may simply be capturing the time elapsed between last coffee drinking and the end of the experiment. Similarly, propionylcarnitine (Reznick et al., 1992, Scioli et al., 2015) was found, like various other carnitines, to decrease after swimming. Since it is related to meat and fish intake (Cheung et al., 2017), its decrease may also be related to the time elapsed since last meal rather than to exposure to DBP.

In addition, we observed increased levels of xanthosine associated with BDCM and total brominated THM and total THM, which is consistent with results from two previous studies (Daskalaki et al., 2015, Lewis et al., 2010). Xanthosine can be degraded to xanthine, which in turn can be converted to uric acid by xanthine oxidase. Polotow et al. observed xanthine oxidase activation and altered levels of uric acid in plasma of trained participants after exercise (one-repetition maximum test with a bench press) and hypothesized that uric acid plays an important role as an antioxidant in plasma (Polotow et al., 2017). Other studies have also observed higher levels of uric acid with lower levels of oxidative stress but higher levels of inflammation (Wu et al., 2015). We observed elevated levels of uric acid after our swimming experiment which could be a response to exercise-induced oxidative stress or due to DBP exposure induced oxidative stress, as Walker et al. similarly identified higher levels of uric acid after occupational TCE exposure (Walker et al., 2016).

Lower levels of cortisol and cortisone were found related to the swimming experiment in this study. These molecules are released in response to stress and suppress the immune system and reduce inflammation. One metabolomic study investigating metabolic changes after physical activity reported lower levels of cortisol (Xiao et al., 2016) but other studies also reported higher hormone levels after exercise (Di Luigi et al., 2014, Sato et al., 2016).

In addition to the endogenous and diet related metabolites, four unidentified chlorinated compounds were found to be associated with swimming. For three compounds, structural analysis could be performed and the MS/MS experiments confirmed the presence of Cl. Several common fragments were observed that correspond to those of compounds containing only C, H, and O atoms, such as unsaturated fatty acids, but the lack of ionization in negative polarity suggested absence of acid functionality. Interestingly, the mass difference of two of the compounds corresponded to that of hypochlorous acid (HOCl), which is the hydration product of Cl_2_ and one of the primary disinfection agents of chlorine solutions. HOCl is reactive with a number of biomolecules *in vivo* and *ex vivo*, including fatty acids, lipids and aromatic amino acids and different chlorinated metabolites are known to form after chlorine exposure (Fu et al., 2000, Hazen et al., 1996, Schroter and Schiller, 2016). The two compounds could thus be two reaction products after an addition of HOCl or Cl_2_ to a double bond of a compound R to yield HO-R-Cl and Cl_2_-R, or the mono- and dichlorinated products of two compounds HO-R and R through aromatic substitution. The relative retention times of the compounds support such identifications (Crow et al., 2016) (see Fig. S6 for details). Although these compounds were also detected in the samples taken before the exposure, their significant increase likely reflects the exposure to the chlorine-containing DBPs in the swimming pool water. In an earlier metabolomic study on occupational exposure to trichloroethylene, it was found that a full shift exposure range of 0.4 to 230 ppm resulted in the detection of several unidentifiable chlorinated metabolites (Walker et al., 2016). None of these however matched by mass with the ones found in the present study. In another recent study a number of chlorinated lipids and their metabolites were discovered in the lungs and plasma of mouse and rat models after chlorine gas exposure (Ford et al., 2016). Other identifiable chlorine adducts include 3-chlorotyrosine and 3,5-dichlorotyrosine that are known to form after chlorine exposure and have been proposed as biomarkers of chlorine exposure (Crow et al., 2016).

Despite the major challenge of disentangling the effect of DBPs from the effect of physical activity, and other experiment-related factors, this study has several strengths. Firstly, contrary to most studies, it assessed more than the usual four trihalomethanes in exhaled air, and additionally included external exposures consisting of various DBPs measured in swimming pool water. Second, physical activity was accurately measured. Finally, the untargeted approach we applied provides unique opportunities for identification of new metabolic features, which once annotated may help identifying affected pathways.

## 5 Conclusions

Metabolomics allowed the identification of several metabolites associated with swimming in a chlorinated swimming pool. However, the high correlation between DBP levels, physical activity during swimming, and the metabolome did not allow a clear separation of the effects of DBPs as such from physical activity. Some metabolic changes appear to be related to physical exercise whereas other changes are more likely attributable to exposure to DBPs.

The following are the supplementary data related to this article.

Fig. S1 Pearson pairwise correlation for DBPs measured in exhaled breath and physical activity (kcal) (A) before the experiment, (B) after the experiment. Estimates are based on 58 observations. Fig. S1

Fig. S2 Pearson pairwise correlation of the exposure measurements. Results are presented for the 7 internal exposure measurements (in exhaled breath and urine), and external exposures (in swimming water). Correlations are given for the measurements obtained after the swimming experiment. Fig. S2

Fig. S3 Pearson pairwise correlation for 293 metabolic features found significantly associated with at least one DBP level measured in exhaled breath using Model 1. Results are presented for metabolomic profiles obtained after the swim. Fig. S3

Fig. S4 Investigating the strength of the associations linking the (*N* = 280) metabolic features found associated with the binary pre-post swimming indicator (Model 1’) in the models regressing metabolic features against the exhaled breath levels of THM (Model 1). The strength of association (*p*-value) for each metabolic feature identified in Model 1’ (X-axis) is compared to that obtained in Model 1 for each exhaled breath exposure measurement (Y-axis). Fig. S4

Fig. S5 Chromatographic peaks and isotopic patterns of the 13 identified metabolites associated with swimming. Isotope peaks (bars) with overlaid theoretical peaks (boxes) calculated for the elemental compositions indicated on top of each spectrum. Fig. S5

Fig. S6 Chromatographic peaks and isotopic patterns and MS/MS spectra of the unidentifiable chlorinated compounds associated with swimming. Isotope peaks (bars) with overlaid theoretical peaks (boxes) calculated for the elemental compositions indicated on top of each spectrum. Fig. S6

Table S1 Details of the 293 metabolic features found associated with at least one exposure level measured in exhaled breath. For each exposure (columns) and each metabolic feature (in rows) we report the strength of association as measured by the p-value, the effect size estimate (β) and the proportion, calculated across the 1000 subsamples, where the association was found significant. For each metabolic feature, we also report the number of associated exposures (# associations, from 1 to 6) and list these exposures. Table S1
