# Supplementary figures and images for: Effects of exposure to water disinfection by-products in a swimming pool: A metabolome-wide association study
Source: Environ Int. 2018 Feb;111:60–70. doi: 10.1016/j.envint.2017.11.017 (PMC5786667; doi:10.1016/j.envint.2017.11.017)

### A– Before Swimming

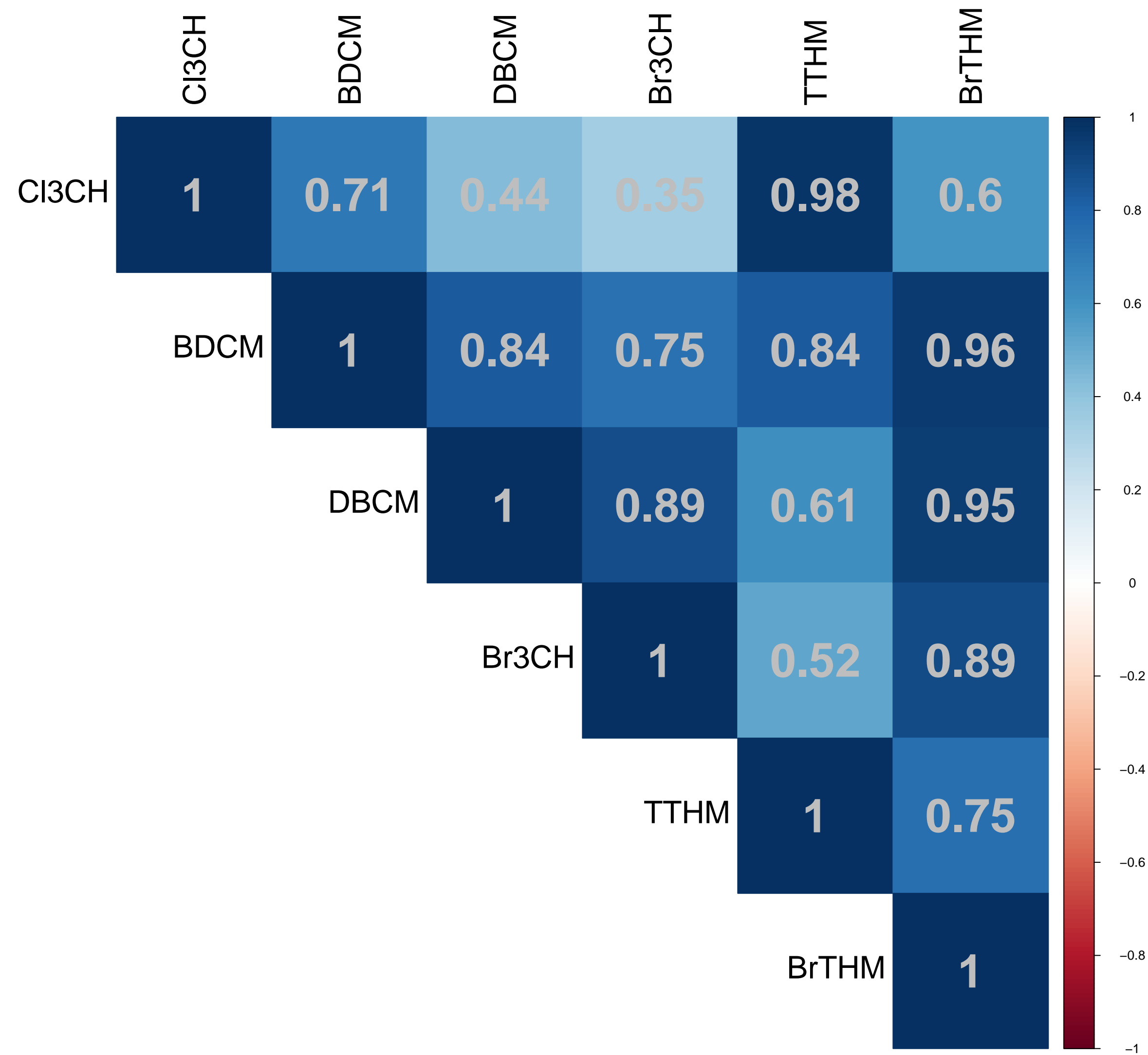

### B– After Swimming

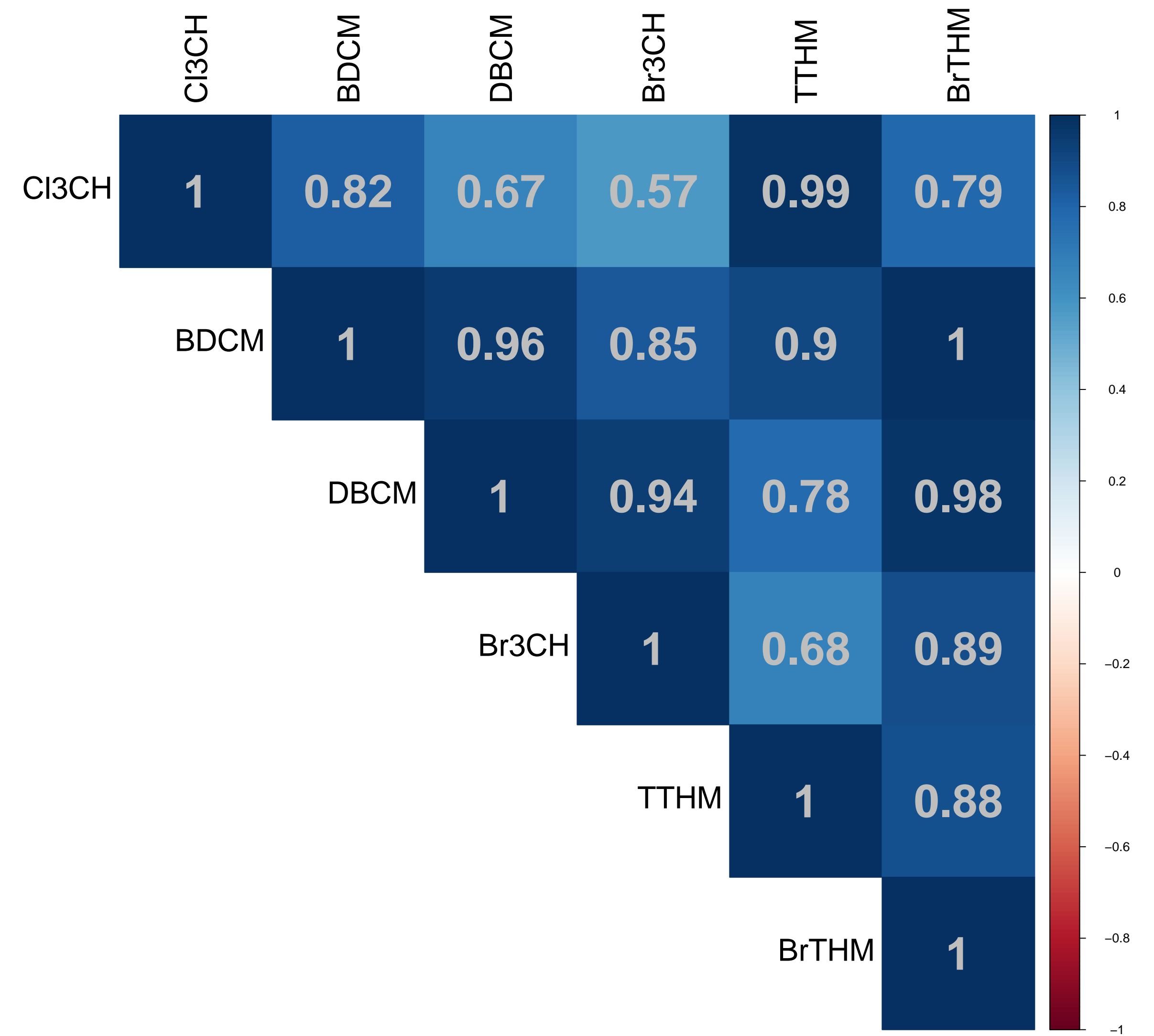

Supplement: Fig. S1 — Pearson pairwise correlation for DBPs measured in exhaled breath and physical activity (kcal) (A) before the experiment, (B) after the experiment. Estimates are based on 58 observations. [file mmc1.pdf]

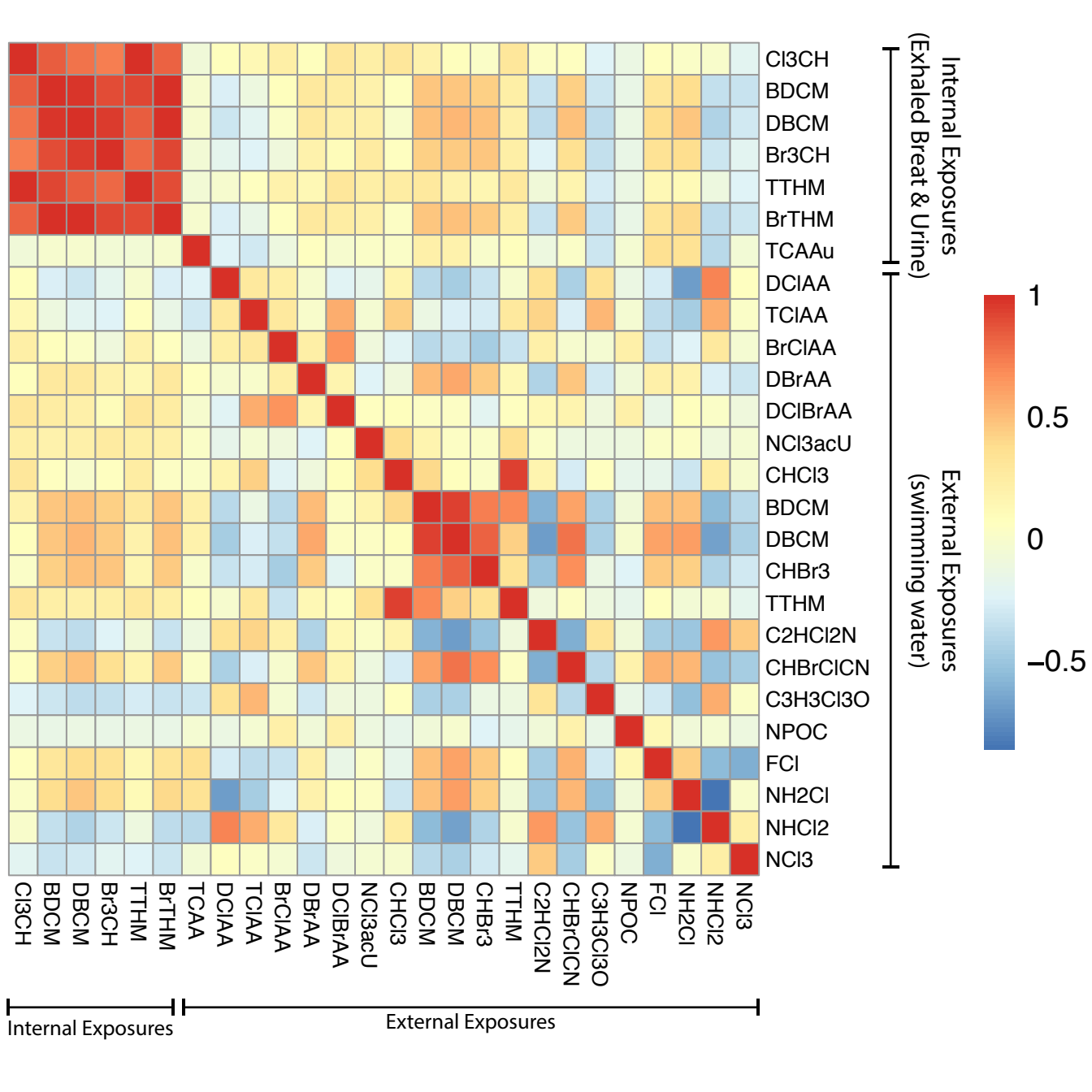

Supplement: Fig. S2 — Pearson pairwise correlation of the exposure measurements. Results are presented for the 7 internal exposure measurements (in exhaled breath and urine), and external exposures (in swimming water). Correlations are given for the measurements obtained after the swimming experiment. [file mmc2.pdf]

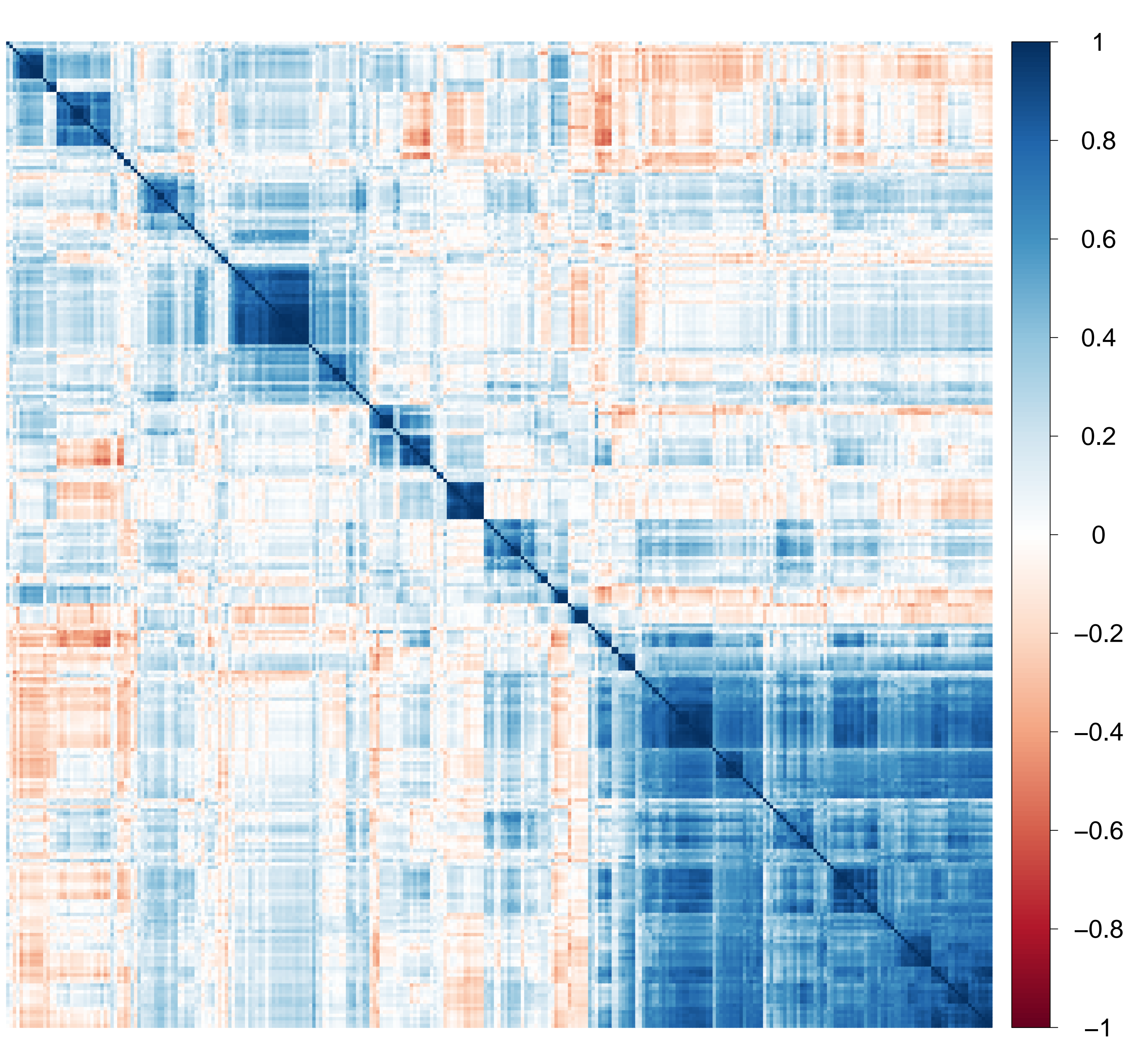

Supplement: Fig. S3 — Pearson pairwise correlation for 293 metabolic features found significantly associated with at least one DBP level measured in exhaled breath using Model 1. Results are presented for metabolomic profiles obtained after the swim. [file mmc3.pdf]

# p-value comparison

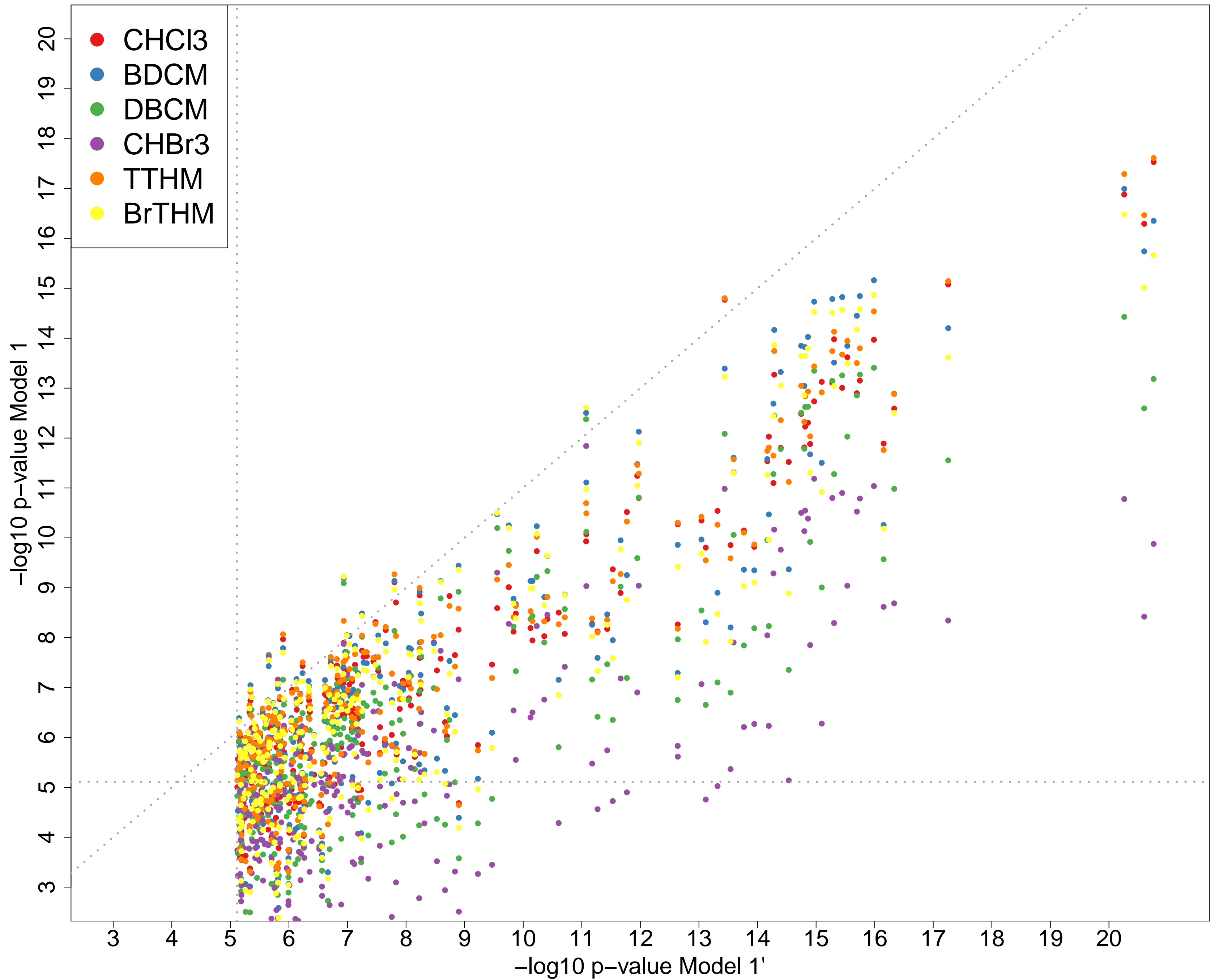

Supplement: Fig. S4 — Investigating the strength of the associations linking the (N = 280) metabolic features found associated with the binary pre-post swimming indicator (Model 1’) in the models regressing metabolic features against the exhaled breath levels of THM (Model 1). The strength of association (p-value) for each metabolic feature identified in Model 1’ (X-axis) is compared to that obtained in Model 1 for each exhaled breath exposure measurement (Y-axis). [file mmc4.pdf]
