# Supplementary material for: Effects of exposure to water disinfection by-products in a swimming pool: A metabolome-wide association study
Source: Environ Int. 2018 Feb;111:60–70. doi: 10.1016/j.envint.2017.11.017 (PMC5786667; doi:10.1016/j.envint.2017.11.017)
Supplement: Fig. S5 — Chromatographic peaks and isotopic patterns of the 13 identified metabolites associated with swimming. Isotope peaks (bars) with overlaid theoretical peaks (boxes) calculated for the elemental compositions indicated on top of each spectrum. [file mmc5.pdf]

Figure S4

Caffeine

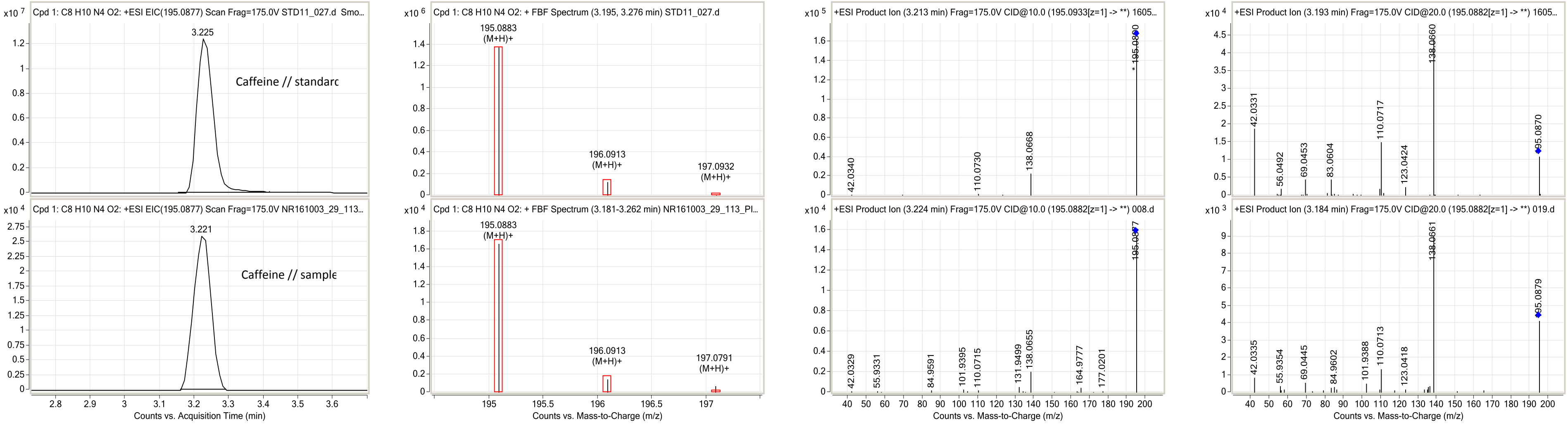

Cortisol

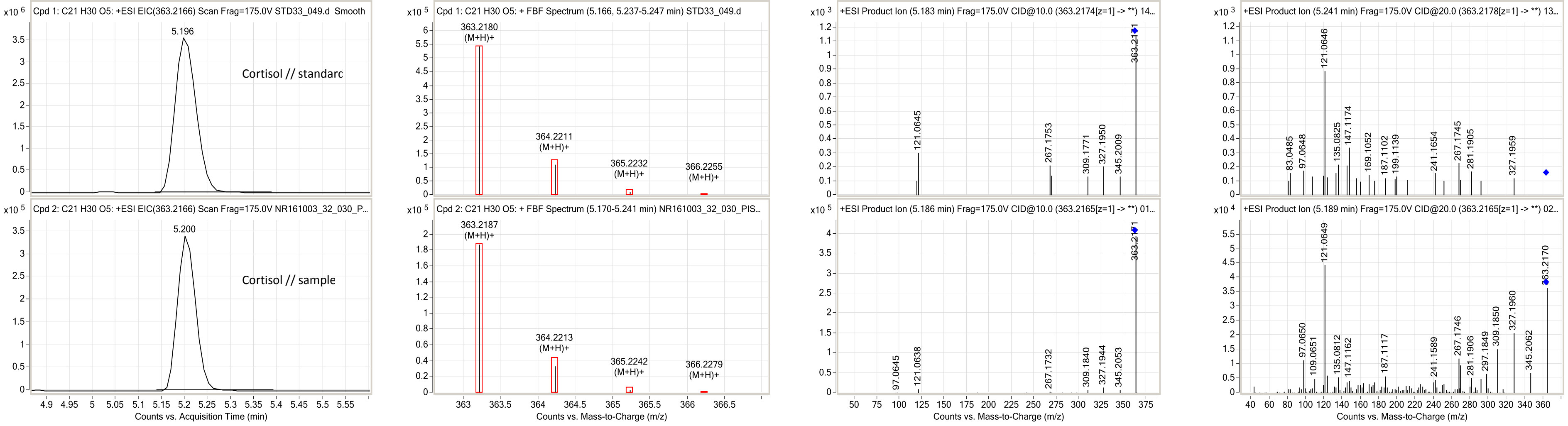

Cortisone

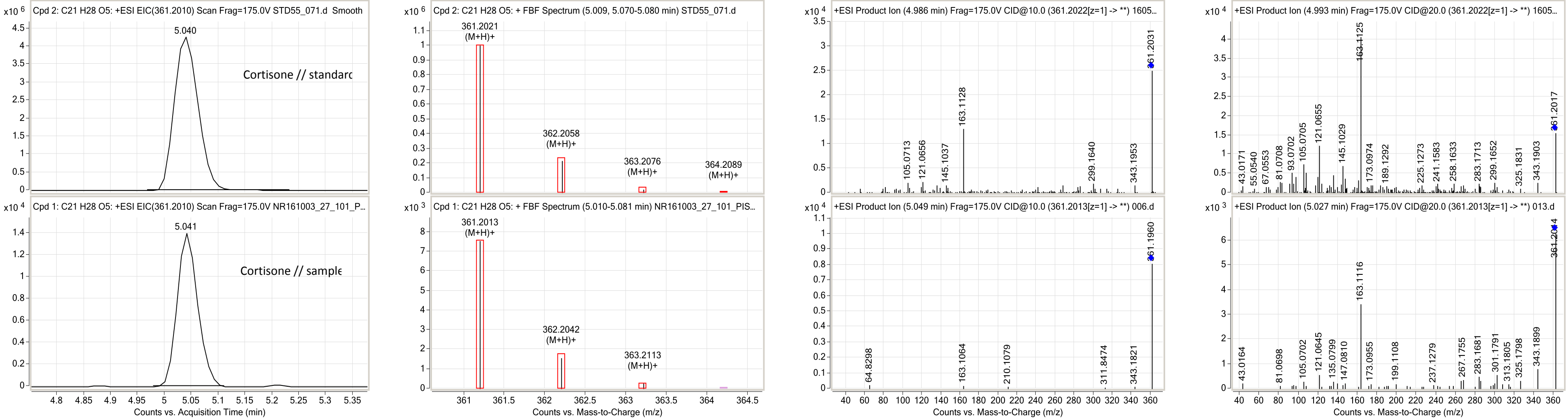

Hydroxyhexanoylcarnitine

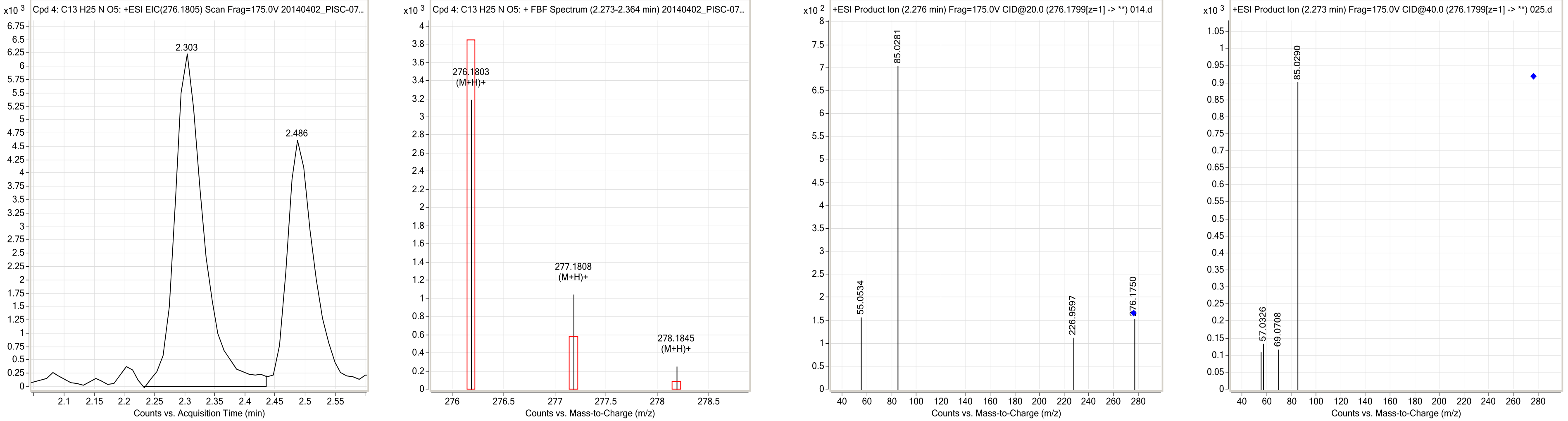

Kynurenine

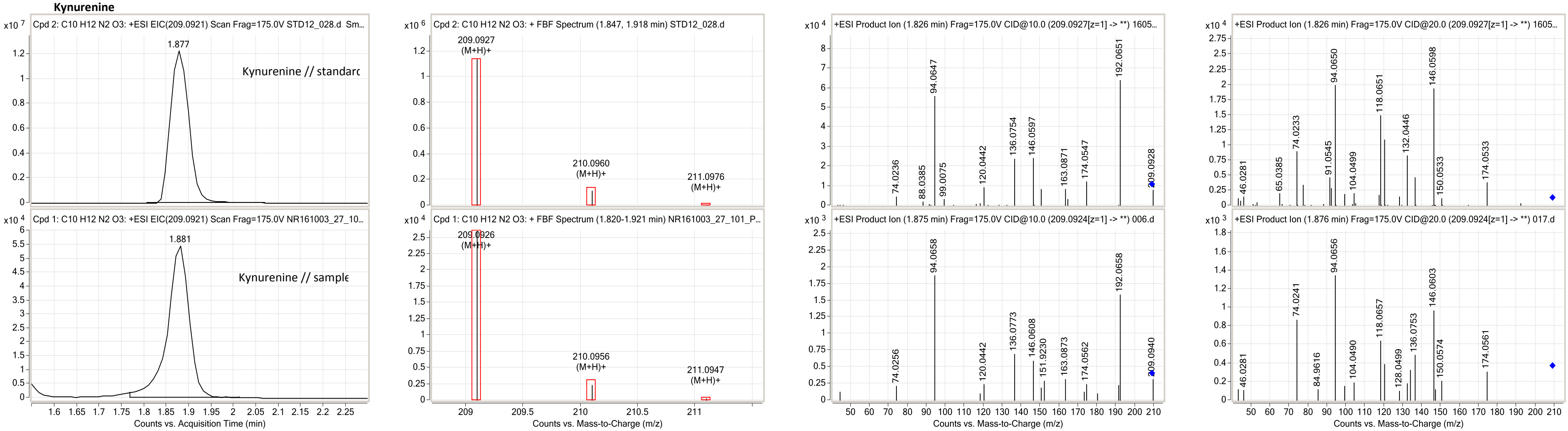

L-gamma-Glutamyl-L-leucine

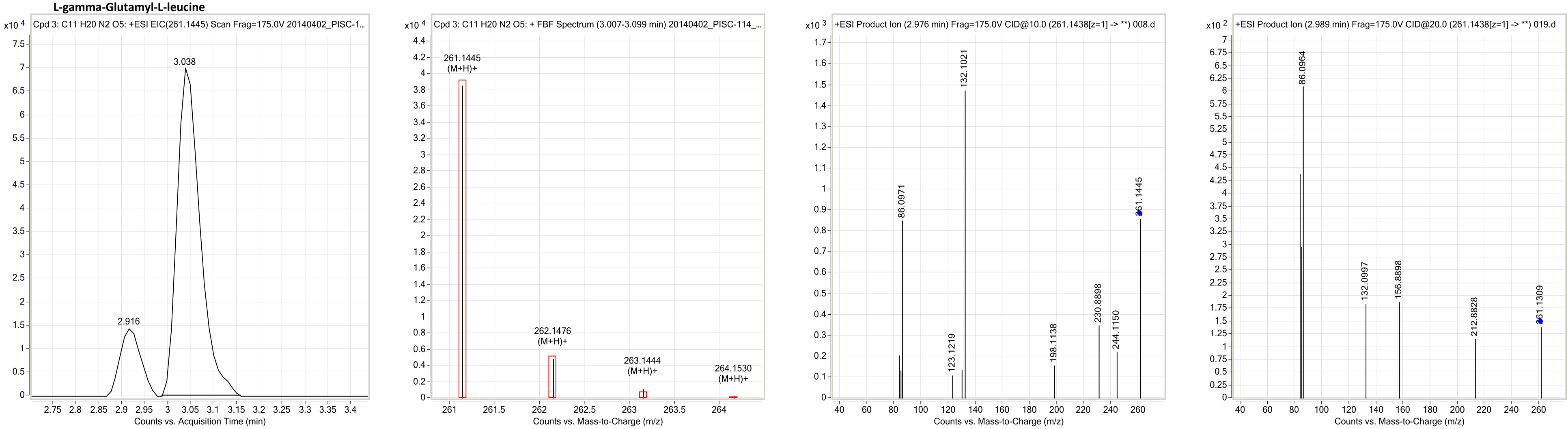

Myristoylcarnitine

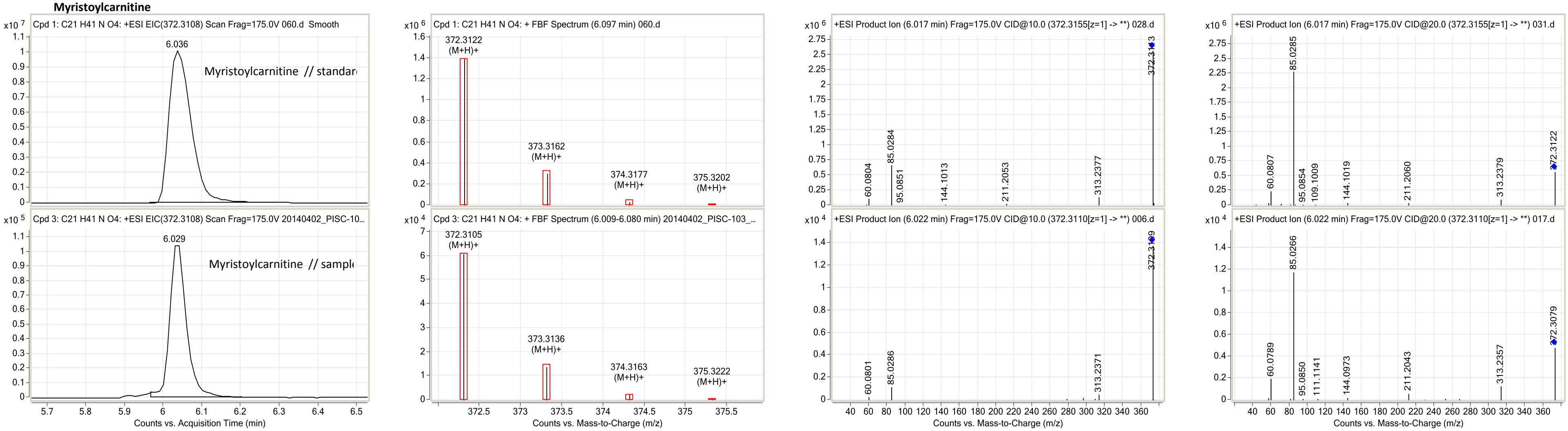

Nonanoylcarnitine

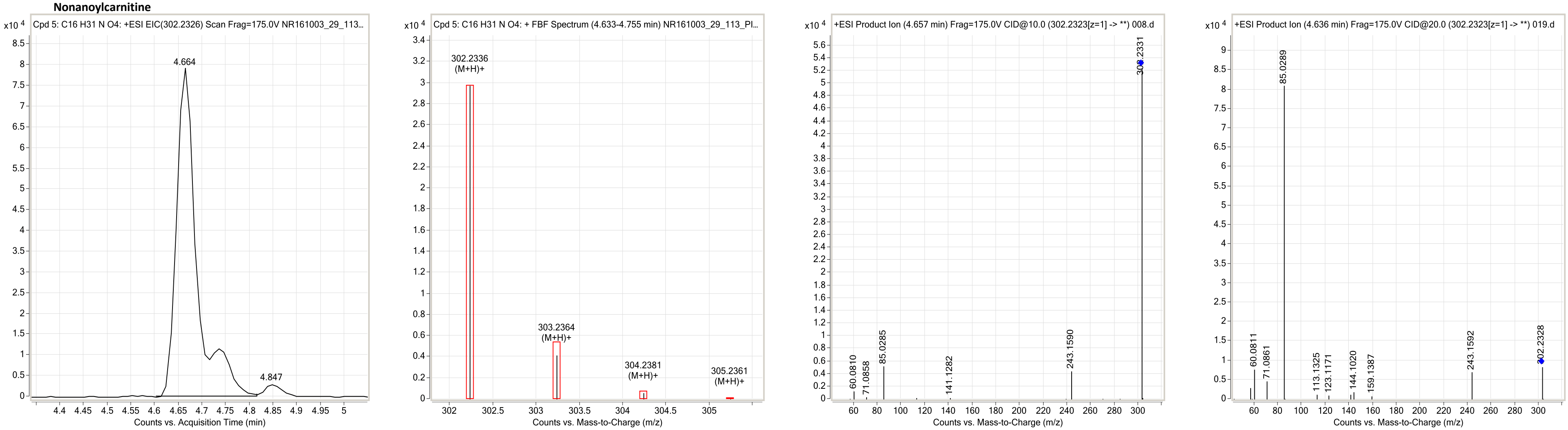

Propionylcarnitine

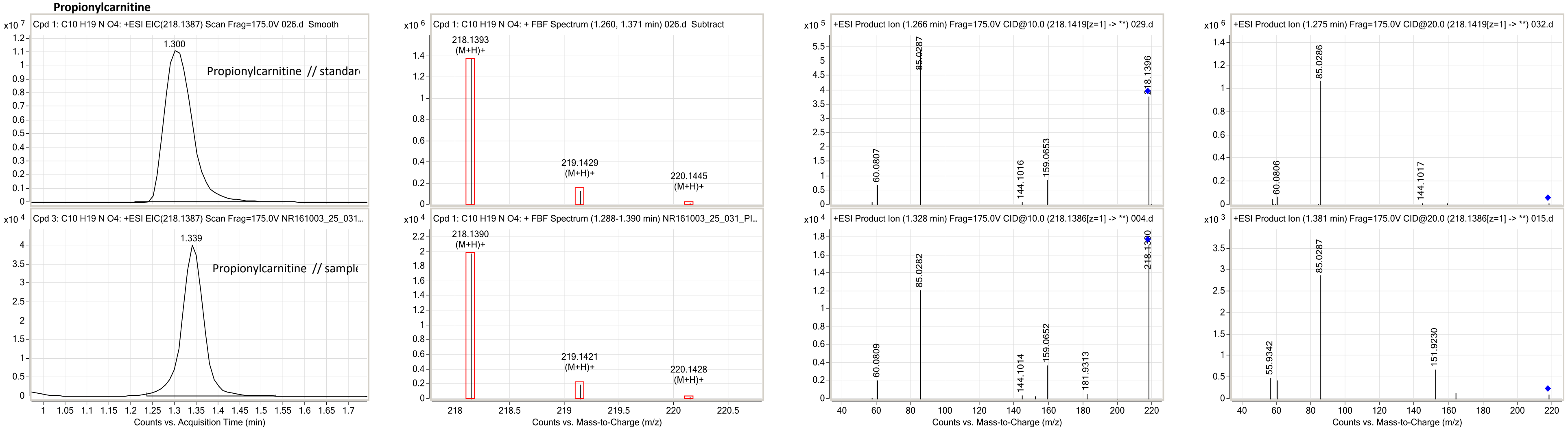

Hexadecenoylcarnitine

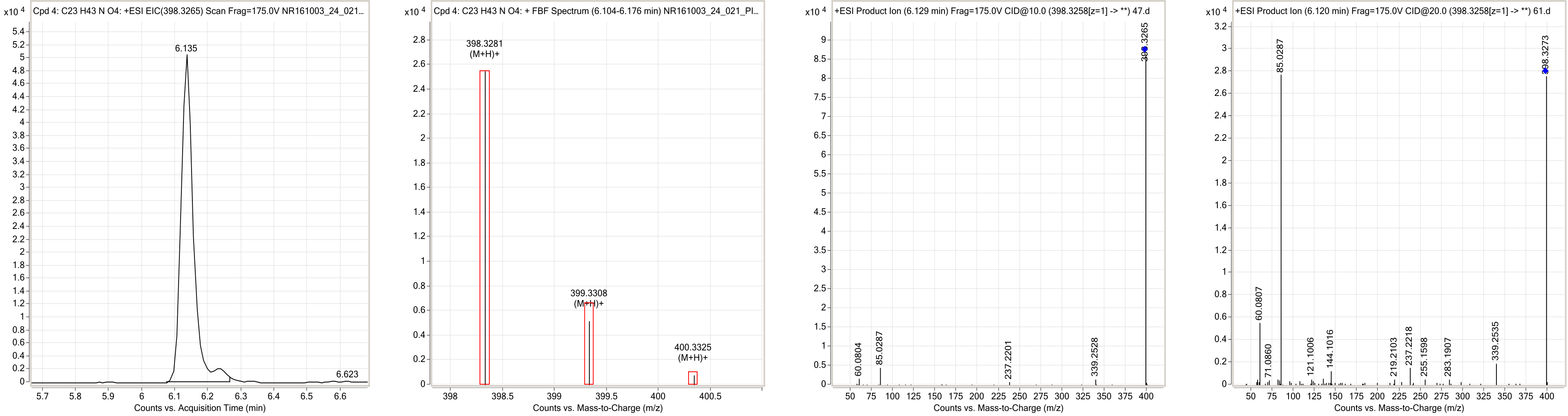

Tryptophan

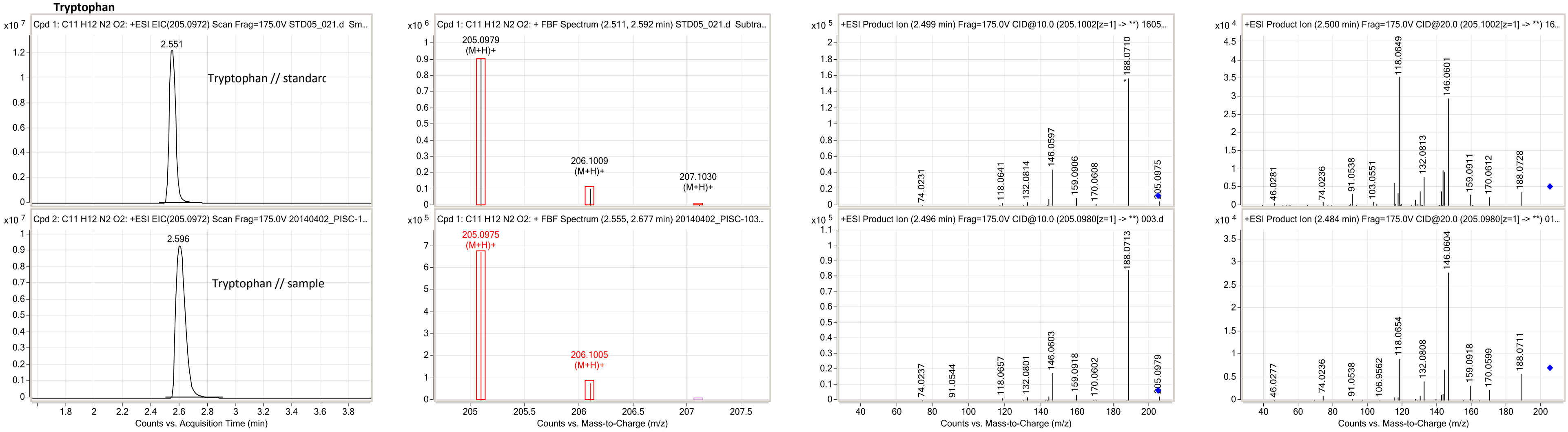

Uric acid

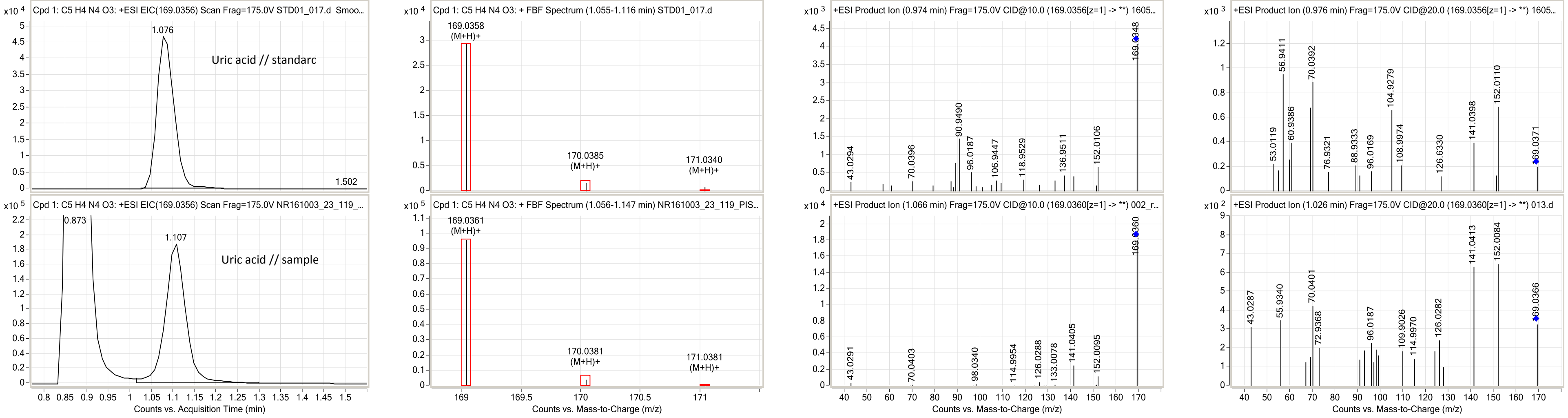

Xanthosine

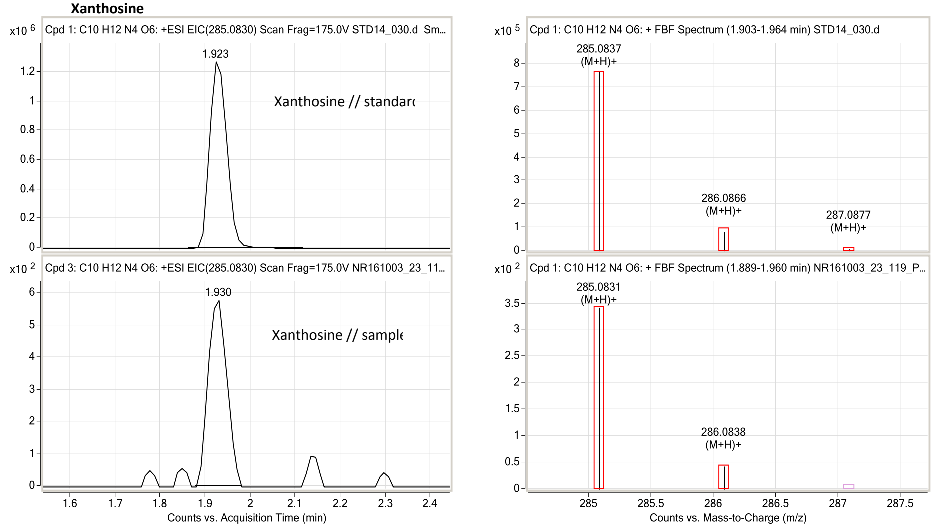

Cpd 1: C10 H12 N4 O6: +ESI EIC(285.0830) Scan Frag=175.0V STD14_030.d Sm...
x10 6
1.923
Xanthosine // standard
Cpd 3: C10 H12 N4 O6: +ESI EIC(285.0830) Scan Frag=175.0V NR161003_23_11...
x10 2
1.930
Xanthosine // sample
Counts vs. Acquisition Time (min)
Cpd 1: C10 H12 N4 O6: + FBF Spectrum (1.903-1.964 min) STD14_030.d
x10 5
285.0837 (M+H)+
286.0866 (M+H)+
287.0877 (M+H)+
Cpd 1: C10 H12 N4 O6: + FBF Spectrum (1.889-1.960 min) NR161003_23_119_P...
x10 2
285.0831 (M+H)+
286.0838 (M+H)+
Counts vs. Mass-to-Charge (m/z)
