# Supplementary material for: Effects of exposure to water disinfection by-products in a swimming pool: A metabolome-wide association study
Source: Environ Int. 2018 Feb;111:60–70. doi: 10.1016/j.envint.2017.11.017 (PMC5786667; doi:10.1016/j.envint.2017.11.017)

Compound 1 (Feature ID: 310.0187@1.8356032)

- No other adducts or neutral losses observed
- Not found in negative ion mode - most likely no anionic functional groups
- Unable to acquire MS/MS spectra
- Best fitting computed formula $C_{13}H_{11}ClN_2O_3S$ (formula generation details as described in methods for finding halogenated features)

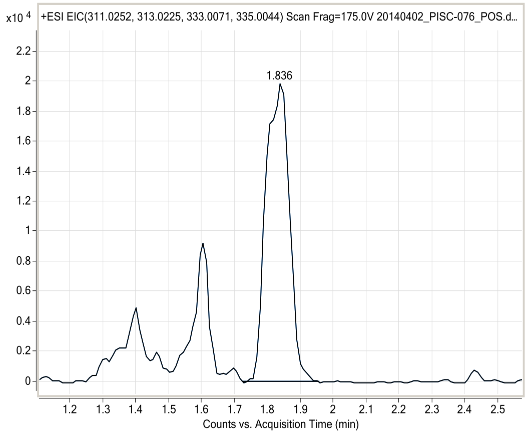

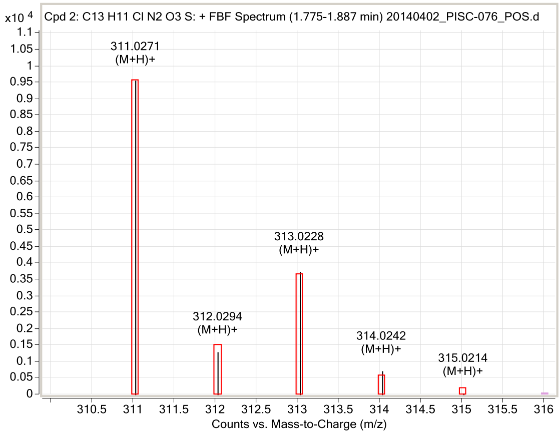

Compound 2 (Feature IDs: 374.1704@4.1407986, 396.1522@4.144505, 396.1523@4.1454406)

- Strong $[M+Na]^+$ (13x) indicates low proton affinity and lack of cationic functional groups
- Not found in negative ion mode - most likely no anionic functional groups
- MS/MS of $[M+H]^+$ shows loss of H_2O with CHO chain fragments *m/z* 43, 57, 99, 173
- MS/MS of $[M+Na]^+$ shows loss of HCl (*m/z* 397.1599 → *m/z* 361.1815), indicating a presence of Cl
- Best-fitting computed formula $C_{15}H_{31}ClO_8$

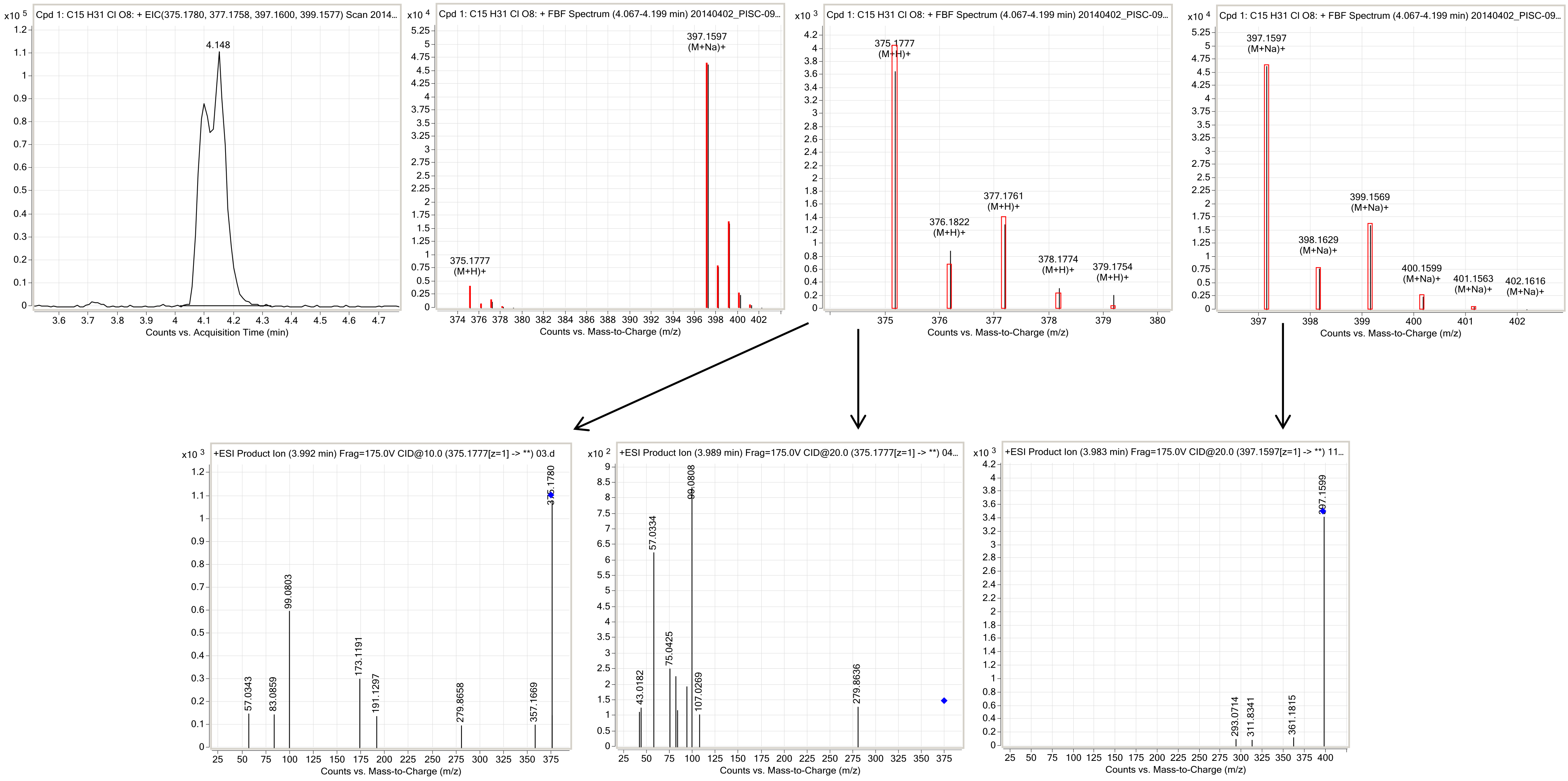

Compound 3 (Feature IDs: 448.2077@4.154416, 470.1892@4.154688)

- Strong $[M+Na]^+$ (19x) indicates low proton affinity and lack of cationic functional groups
- Not found in negative ion mode - most likely no anionic functional groups
- MS/MS of $[M+H]^+$ shows CHO chain fragments *m/z* 43, 57, 99, 173
- MS/MS of $[M+Na]^+$ shows CHO chain fragments *m/z* 57, 99 and loss of HCl (*m/z* 471.1962 → *m/z* 435.2198), indicating a presence of Cl
- Best-fitting computed formula $C_{18}H_{37}ClO_{10}$

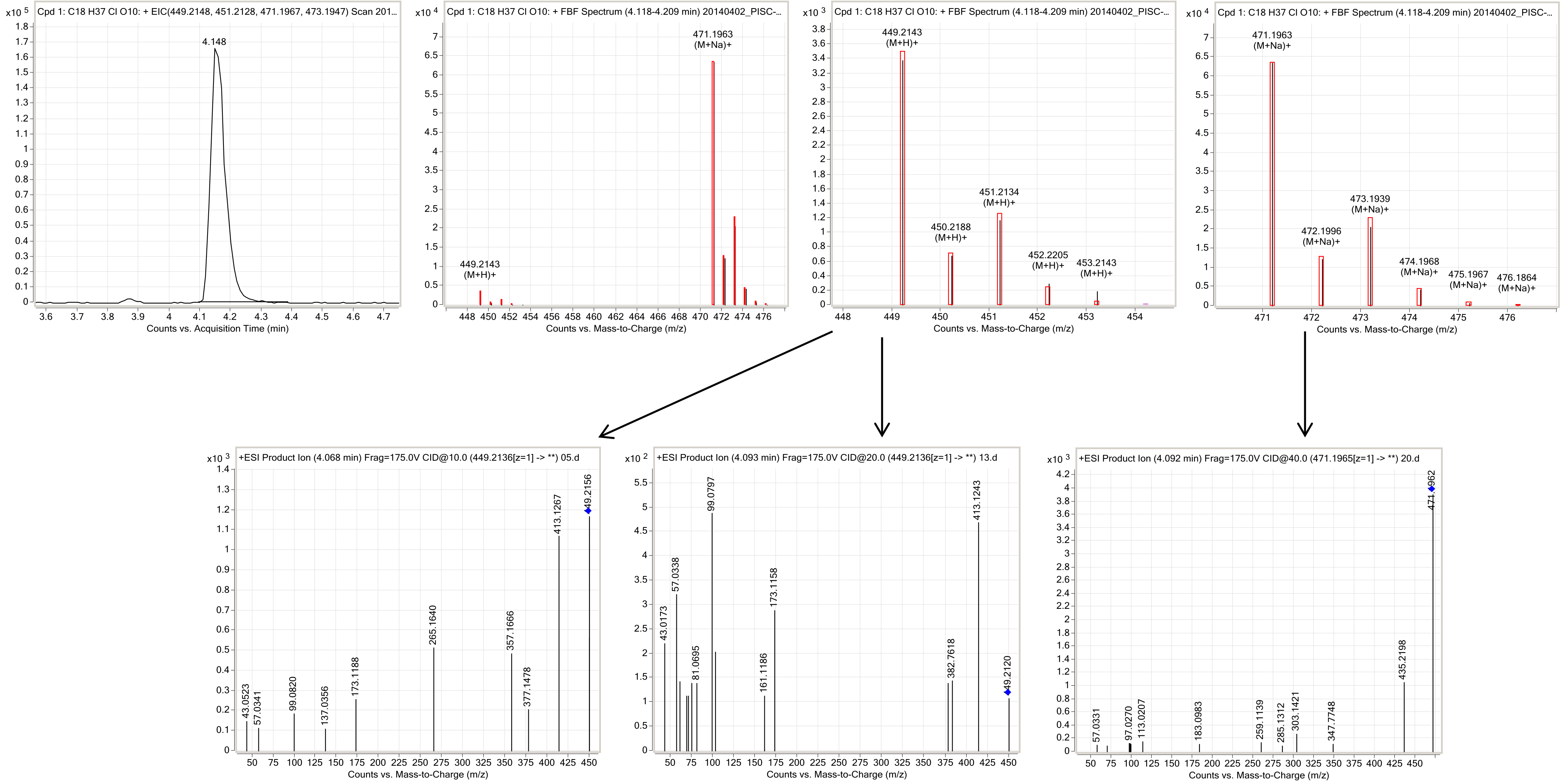

Compound 4 (Feature ID: 488.1561@4.923312)

- Strong ionization as $[M+Na]^+$ indicates low proton affinity and lack of cationic functional groups
- Not found in negative ion mode - most likely no anionic functional groups
- MS/MS of $[M+H]^+$ shows CHO chain fragments *m/z* 57, 99
- MS/MS of $[M+Na]^+$ shows CHO chain fragments *m/z* 57, 99 and loss of HCl (m/z 489.1619 > m/z 453.1857), indicating a presence of Cl
- Best-fitting computed formula: $C_{18}H_{36}Cl_2O_9$

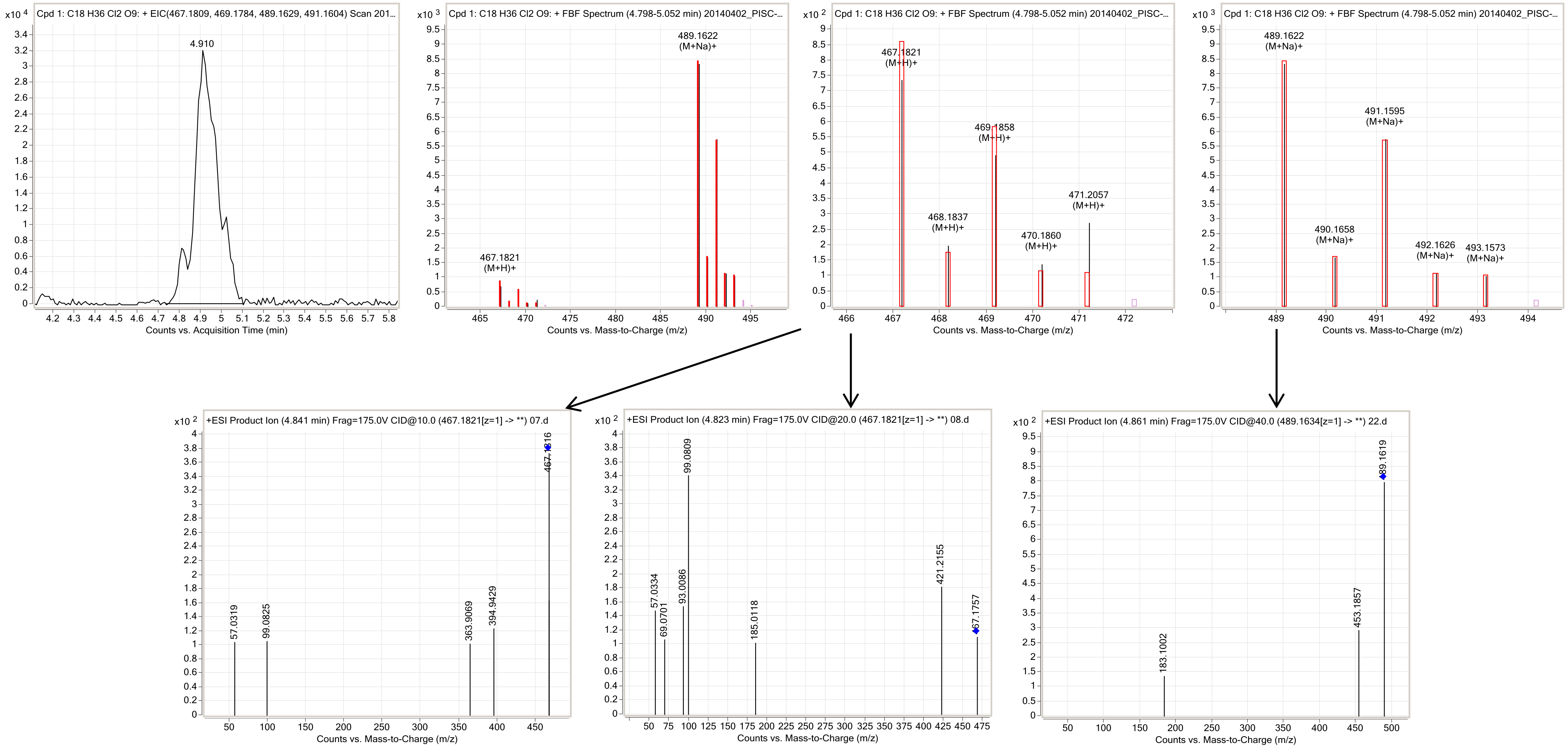

Supplement: Fig. S6 — Chromatographic peaks and isotopic patterns and MS/MS spectra of the unidentifiable chlorinated compounds associated with swimming. Isotope peaks (bars) with overlaid theoretical peaks (boxes) calculated for the elemental compositions indicated on top of each spectrum. [file mmc6.pdf]
