# Supplementary material for: Effects of exposure to water disinfection by-products in a swimming pool: A metabolome-wide association study
Source: Environ Int. 2018 Feb;111:60–70. doi: 10.1016/j.envint.2017.11.017 (PMC5786667; doi:10.1016/j.envint.2017.11.017)
Supplement: Table S1 — Details of the 293 metabolic features found associated with at least one exposure level measured in exhaled breath. For each exposure (columns) and each metabolic feature (in rows) we report the strength of association as measured by the p-value, the effect size estimate (β) and the proportion, calculated across the 1000 subsamples, where the association was found significant. For each metabolic feature, we also report the number of associated exposures (# associations, from 1 to 6) and list these exposures. [file mmc7.pdf]

Table S1

Feature ID (m@t)	CHCl3		BDCM		DBCM		CHBr3		TTHM		BrTHM		# Associati	Associated exposures
	p-value	b	p-value	b	p-value	b	p-value	b	p-value	b	p-value	b		
168.0287@1.1498789	1.98E-06	0.03	3.24E-07	0.15	5.70E-07	0.64	2.07E-06	3.21	1.07E-06	0.02	3.31E-07	0.12	6	CHCl3 BDCM DBCM CHBr3 TTHM BrTHM
217.1313@1.361564	1.51E-10	-0.06	4.47E-10	-0.24	6.48E-09	-1.04	5.36E-07	-4.88	1.36E-10	-0.04	7.84E-10	-0.19	6	CHCl3 BDCM DBCM CHBr3 TTHM BrTHM
261.0047@1.3606347	2.54E-08	0.07	1.25E-08	0.32	1.19E-07	1.37	2.04E-06	6.55	1.70E-08	0.06	1.94E-08	0.25	6	CHCl3 BDCM DBCM CHBr3 TTHM BrTHM
202.9641@1.3599007	2.44E-08	0.07	3.29E-09	0.34	2.36E-08	1.46	3.12E-07	6.96	1.21E-08	0.06	4.58E-09	0.27	6	CHCl3 BDCM DBCM CHBr3 TTHM BrTHM
205.974@1.3634889	2.47E-07	0.07	7.37E-08	0.32	3.97E-07	1.38	2.59E-06	6.66	1.52E-07	0.06	9.75E-08	0.25	6	CHCl3 BDCM DBCM CHBr3 TTHM BrTHM
202.9645@1.6236619	3.29E-09	0.08	2.10E-09	0.36	4.69E-08	1.53	2.81E-06	7.02	2.32E-09	0.06	4.09E-09	0.29	6	CHCl3 BDCM DBCM CHBr3 TTHM BrTHM
175.0441@1.828929	1.27E-09	0.07	1.12E-10	0.33	9.50E-10	1.41	6.56E-08	6.75	5.30E-10	0.06	1.66E-10	0.26	6	CHCl3 BDCM DBCM CHBr3 TTHM BrTHM
224.9633@1.8346719	6.66E-07	0.06	1.63E-07	0.3	4.80E-07	1.31	1.49E-06	6.58	3.90E-07	0.05	1.86E-07	0.24	6	CHCl3 BDCM DBCM CHBr3 TTHM BrTHM
307.0106@1.8356434	1.85E-10	0.08	5.84E-11	0.35	6.06E-10	1.51	5.87E-09	7.61	9.50E-11	0.06	8.31E-11	0.28	6	CHCl3 BDCM DBCM CHBr3 TTHM BrTHM
126.0289@1.8320644	1.12E-07	0.07	5.62E-08	0.3	5.27E-07	1.25	4.97E-06	6.1	7.81E-08	0.05	8.69E-08	0.23	6	CHCl3 BDCM DBCM CHBr3 TTHM BrTHM
238.0012@1.8354497	4.66E-08	0.08	9.03E-09	0.35	4.00E-08	1.51	2.97E-07	7.42	2.46E-08	0.06	1.12E-08	0.28	6	CHCl3 BDCM DBCM CHBr3 TTHM BrTHM
284.9724@1.8343358	5.37E-14	0.09	6.81E-15	0.39	3.54E-13	1.69	6.82E-11	8.27	1.80E-14	0.07	1.36E-14	0.31	6	CHCl3 BDCM DBCM CHBr3 TTHM BrTHM
203.972@1.8368001	7.91E-14	0.08	1.63E-15	0.39	7.11E-14	1.68	1.59E-11	8.26	1.81E-14	0.07	3.07E-15	0.31	6	CHCl3 BDCM DBCM CHBr3 TTHM BrTHM
234.9906@1.8370998	7.05E-14	0.08	1.43E-15	0.39	5.35E-14	1.68	1.63E-11	8.24	1.59E-14	0.07	2.61E-15	0.31	6	CHCl3 BDCM DBCM CHBr3 TTHM BrTHM
202.9648@1.8355144	4.92E-13	0.08	9.42E-15	0.38	2.36E-13	1.66	4.12E-11	8.14	1.17E-13	0.07	1.59E-14	0.3	6	CHCl3 BDCM DBCM CHBr3 TTHM BrTHM
188.9857@1.8333958	2.63E-08	0.07	7.32E-10	0.33	1.64E-09	1.45	1.83E-08	7.21	8.91E-09	0.06	7.61E-10	0.26	6	CHCl3 BDCM DBCM CHBr3 TTHM BrTHM
292.0468@1.8352855	1.26E-13	0.08	3.55E-15	0.39	1.40E-13	1.67	2.98E-11	8.21	3.13E-14	0.07	6.62E-15	0.31	6	CHCl3 BDCM DBCM CHBr3 TTHM BrTHM
264.014@1.8356805	1.57E-12	0.08	4.74E-14	0.39	1.65E-12	1.66	1.73E-10	8.13	4.39E-13	0.07	8.83E-14	0.3	6	CHCl3 BDCM DBCM CHBr3 TTHM BrTHM
261.0058@1.8364325	1.84E-13	0.08	1.85E-15	0.39	4.48E-14	1.69	6.54E-12	8.34	3.66E-14	0.07	2.98E-15	0.31	6	CHCl3 BDCM DBCM CHBr3 TTHM BrTHM
259.0096@1.83347	1.17E-10	0.08	3.14E-13	0.37	4.21E-13	1.65	1.44E-12	8.5	2.02E-11	0.06	2.43E-13	0.29	6	CHCl3 BDCM DBCM CHBr3 TTHM BrTHM
310.0187@1.8356032	8.49E-11	0.08	7.76E-12	0.35	7.55E-11	1.51	9.25E-10	7.57	3.22E-11	0.06	1.06E-11	0.28	6	CHCl3 BDCM DBCM CHBr3 TTHM BrTHM
206.0372@1.8375108	5.91E-13	0.08	1.51E-14	0.38	2.39E-13	1.66	2.84E-11	8.18	1.46E-13	0.07	2.25E-14	0.3	6	CHCl3 BDCM DBCM CHBr3 TTHM BrTHM
232.053@1.8346452	1.54E-12	0.08	9.05E-14	0.37	1.64E-12	1.58	7.34E-11	7.85	4.73E-13	0.06	1.39E-13	0.29	6	CHCl3 BDCM DBCM CHBr3 TTHM BrTHM
221.0111@1.8376855	3.28E-13	0.08	1.42E-14	0.38	3.12E-13	1.67	3.15E-11	8.23	8.98E-14	0.07	2.28E-14	0.3	6	CHCl3 BDCM DBCM CHBr3 TTHM BrTHM
256.019@1.8363427	9.86E-14	0.08	1.49E-15	0.39	5.55E-14	1.7	1.26E-11	8.33	2.12E-14	0.07	2.69E-15	0.31	6	CHCl3 BDCM DBCM CHBr3 TTHM BrTHM
178.042@1.8345565	9.76E-10	0.07	5.62E-11	0.31	1.82E-10	1.38	5.28E-09	6.79	3.50E-10	0.05	6.32E-11	0.25	6	CHCl3 BDCM DBCM CHBr3 TTHM BrTHM
191.0579@1.9298067	2.99E-08	-0.04	6.03E-08	-0.19	1.26E-07	-0.85	3.02E-07	-4.35	2.52E-08	-0.03	6.19E-08	-0.15	6	CHCl3 BDCM DBCM CHBr3 TTHM BrTHM
208.0851@1.9304636	5.26E-08	-0.04	1.78E-07	-0.2	5.16E-07	-0.85	1.48E-06	-4.27	5.22E-08	-0.04	2.01E-07	-0.15	6	CHCl3 BDCM DBCM CHBr3 TTHM BrTHM
284.0759@1.9795315	1.25E-08	0.08	1.30E-08	0.34	1.03E-07	1.43	7.25E-07	7.08	9.52E-09	0.06	1.85E-08	0.26	6	CHCl3 BDCM DBCM CHBr3 TTHM BrTHM
275.1728@2.306461	6.91E-09	0.04	3.60E-10	0.21	1.20E-09	0.91	6.85E-08	4.38	2.62E-09	0.04	4.34E-10	0.16	6	CHCl3 BDCM DBCM CHBr3 TTHM BrTHM
275.1724@2.4903276	7.56E-09	0.04	1.66E-09	0.2	5.99E-09	0.88	2.88E-07	4.19	3.90E-09	0.03	2.07E-09	0.16	6	CHCl3 BDCM DBCM CHBr3 TTHM BrTHM
408.1793@2.5994506	1.78E-07	-0.05	3.88E-07	-0.24	1.72E-06	-1.01	5.64E-06	-5.06	1.70E-07	-0.04	4.97E-07	-0.19	6	CHCl3 BDCM DBCM CHBr3 TTHM BrTHM
204.0907@2.5986507	2.73E-07	-0.05	5.10E-07	-0.24	2.14E-06	-1.01	7.11E-06	-5.04	2.53E-07	-0.04	6.47E-07	-0.19	6	CHCl3 BDCM DBCM CHBr3 TTHM BrTHM
129.0577@2.6053708	1.09E-06	-0.04	7.03E-07	-0.19	1.71E-06	-0.8	7.19E-06	-3.98	7.96E-07	-0.03	7.88E-07	-0.15	6	CHCl3 BDCM DBCM CHBr3 TTHM BrTHM
325.0229@3.2997513	5.44E-09	-0.01	5.10E-08	-0.05	1.78E-07	-0.23	1.47E-06	-1.15	6.62E-09	-0.01	6.19E-08	-0.04	6	CHCl3 BDCM DBCM CHBr3 TTHM BrTHM
194.0809@3.2998073	1.29E-12	-0.01	5.54E-11	-0.05	2.69E-10	-0.23	2.41E-09	-1.13	1.74E-12	-0.01	6.52E-11	-0.04	6	CHCl3 BDCM DBCM CHBr3 TTHM BrTHM
303.2048@3.818443	1.64E-07	0.05	1.61E-08	0.22	6.40E-08	0.94	1.63E-06	4.46	7.85E-08	0.04	2.07E-08	0.17	6	CHCl3 BDCM DBCM CHBr3 TTHM BrTHM
396.1523@4.1454406	1.32E-17	0.08	1.01E-17	0.35	3.72E-15	1.5	1.67E-11	7.13	5.12E-18	0.06	3.32E-17	0.28	6	CHCl3 BDCM DBCM CHBr3 TTHM BrTHM
470.1892@4.154688	9.37E-13	0.07	3.39E-11	0.29	5.85E-09	1.17	5.87E-07	5.47	1.55E-12	0.05	1.09E-10	0.22	6	CHCl3 BDCM DBCM CHBr3 TTHM BrTHM
488.1561@4.923312	2.57E-13	0.09	1.28E-13	0.4	1.04E-11	1.68	2.05E-09	8.1	1.31E-13	0.07	3.13E-13	0.31	6	CHCl3 BDCM DBCM CHBr3 TTHM BrTHM
360.1939@5.104424	1.40E-10	-0.08	6.24E-09	-0.31	1.26E-07	-1.31	4.32E-06	-6.16	2.57E-10	-0.06	1.19E-08	-0.25	6	CHCl3 BDCM DBCM CHBr3 TTHM BrTHM
429.1004@5.2514434	2.40E-14	0.09	1.42E-14	0.4	9.36E-13	1.71	9.10E-10	8	1.13E-14	0.07	3.19E-14	0.31	6	CHCl3 BDCM DBCM CHBr3 TTHM BrTHM
393.2147@5.2511854	2.91E-12	0.08	2.64E-12	0.37	1.11E-10	1.56	8.99E-09	7.55	1.80E-12	0.06	5.53E-12	0.29	6	CHCl3 BDCM DBCM CHBr3 TTHM BrTHM
376.1886@5.25103	4.49E-11	0.08	1.08E-10	0.34	2.85E-09	1.45	8.56E-08	7.06	3.77E-11	0.06	2.07E-10	0.27	6	CHCl3 BDCM DBCM CHBr3 TTHM BrTHM
476.1125@5.2508264	4.90E-12	0.08	2.47E-12	0.37	8.70E-11	1.57	1.25E-08	7.52	2.67E-12	0.06	4.99E-12	0.29	6	CHCl3 BDCM DBCM CHBr3 TTHM BrTHM
460.1393@5.2541914	7.13E-11	0.07	4.34E-10	0.33	1.43E-08	1.36	6.19E-07	6.5	7.84E-11	0.06	9.21E-10	0.25	6	CHCl3 BDCM DBCM CHBr3 TTHM BrTHM
421.2826@5.2636666	8.80E-08	-0.06	5.04E-08	-0.29	1.80E-07	-1.25	1.64E-06	-6.12	5.98E-08	-0.05	6.16E-08	-0.23	6	CHCl3 BDCM DBCM CHBr3 TTHM BrTHM
362.2092@5.263123	6.68E-09	-0.07	3.42E-09	-0.32	3.41E-08	-1.37	1.81E-06	-6.44	4.39E-09	-0.06	5.54E-09	-0.25	6	CHCl3 BDCM DBCM CHBr3 TTHM BrTHM
273.1756@5.3126297	2.29E-07	0.05	1.69E-08	0.24	5.81E-08	1.05	4.05E-06	4.85	1.05E-07	0.04	2.21E-08	0.19	6	CHCl3 BDCM DBCM CHBr3 TTHM BrTHM
324.1333@5.334951	2.94E-18	0.1	4.42E-17	0.42	6.54E-14	1.77	1.32E-10	8.43	2.45E-18	0.08	2.16E-16	0.33	6	CHCl3 BDCM DBCM CHBr3 TTHM BrTHM
275.1927@5.5056496	1.13E-08	0.05	7.33E-10	0.25	3.68E-09	1.1	3.15E-07	5.18	4.68E-09	0.04	1.00E-09	0.2	6	CHCl3 BDCM DBCM CHBr3 TTHM BrTHM
301.207@5.742206	6.42E-09	0.05	7.32E-10	0.22	4.22E-09	0.96	3.98E-07	4.49	2.95E-09	0.04	1.04E-09	0.17	6	CHCl3 BDCM DBCM CHBr3 TTHM BrTHM
321.136@6.510958	5.32E-11	0.08	1.38E-10	0.34	1.08E-08	1.41	2.43E-06	6.37	4.97E-11	0.06	3.79E-10	0.27	6	CHCl3 BDCM DBCM CHBr3 TTHM BrTHM
414.2733@6.8224254	1.57E-06	-0.05	2.44E-06	-0.2	4.29E-06	-0.88	6.76E-06	-4.47	1.37E-06	-0.04	2.49E-06	-0.16	6	CHCl3 BDCM DBCM CHBr3 TTHM BrTHM
409.317@6.8256383	1.37E-07	-0.05	4.91E-07	-0.22	1.27E-06	-0.94	2.43E-06	-4.81	1.40E-07	-0.04	5.39E-07	-0.17	6	CHCl3 BDCM DBCM CHBr3 TTHM BrTHM

525.2894@6.9272556	3.71E-08	0.06	5.15E-08	0.28	3.27E-07	1.2	7.46E-06	5.64	3.12E-08	0.05	7.46E-08	0.22	6	CHCl3 BDCM DBCM CHBr3 TTHM BrTHM
522.7937@7.0420556	8.38E-09	-0.04	1.37E-09	-0.19	2.68E-09	-0.82	3.84E-08	-4.05	3.92E-09	-0.03	1.40E-09	-0.15	6	CHCl3 BDCM DBCM CHBr3 TTHM BrTHM
298.1826@7.1718454	9.28E-07	0.07	3.20E-07	0.3	1.09E-06	1.29	6.92E-06	6.33	5.98E-07	0.05	3.90E-07	0.24	6	CHCl3 BDCM DBCM CHBr3 TTHM BrTHM
354.1482@7.237959	6.76E-07	0.07	2.41E-07	0.32	9.41E-07	1.36	4.22E-06	6.73	4.38E-07	0.05	2.96E-07	0.25	6	CHCl3 BDCM DBCM CHBr3 TTHM BrTHM
308.1444@7.237903	1.44E-06	0.07	4.14E-07	0.33	1.26E-06	1.41	6.18E-06	6.94	8.96E-07	0.06	4.84E-07	0.26	6	CHCl3 BDCM DBCM CHBr3 TTHM BrTHM
273.1666@7.2781734	3.47E-07	-0.05	1.26E-07	-0.25	4.16E-07	-1.08	2.15E-06	-5.33	2.19E-07	-0.04	1.48E-07	-0.2	6	CHCl3 BDCM DBCM CHBr3 TTHM BrTHM
328.2424@7.273751	3.71E-06	0.06	4.33E-07	0.28	6.39E-07	1.25	1.71E-06	6.31	1.89E-06	0.05	4.25E-07	0.22	6	CHCl3 BDCM DBCM CHBr3 TTHM BrTHM
550.8251@7.2789655	3.23E-06	-0.03	4.66E-07	-0.14	3.29E-07	-0.63	2.70E-07	-3.31	1.64E-06	-0.02	3.76E-07	-0.11	6	CHCl3 BDCM DBCM CHBr3 TTHM BrTHM
350.225@7.2749085	4.12E-06	0.06	7.85E-07	0.28	1.30E-06	1.23	3.37E-06	6.21	2.34E-06	0.05	7.93E-07	0.22	6	CHCl3 BDCM DBCM CHBr3 TTHM BrTHM
283.2869@7.4410996	1.22E-06	0.07	4.11E-07	0.31	1.17E-06	1.35	3.91E-06	6.77	7.78E-07	0.05	4.69E-07	0.25	6	CHCl3 BDCM DBCM CHBr3 TTHM BrTHM
405.2297@7.4434114	2.42E-08	0.07	3.16E-08	0.31	2.05E-07	1.31	2.05E-06	6.37	1.98E-08	0.05	4.43E-08	0.24	6	CHCl3 BDCM DBCM CHBr3 TTHM BrTHM
340.1919@7.4432306	8.56E-08	0.07	1.40E-07	0.32	8.68E-07	1.35	7.49E-06	6.54	7.64E-08	0.06	1.97E-07	0.25	6	CHCl3 BDCM DBCM CHBr3 TTHM BrTHM
310.1589@7.443798	7.05E-09	0.08	1.30E-08	0.33	9.91E-08	1.41	1.37E-06	6.82	6.10E-09	0.06	1.90E-08	0.26	6	CHCl3 BDCM DBCM CHBr3 TTHM BrTHM
342.2621@7.4437137	2.35E-08	0.07	3.26E-08	0.31	2.34E-07	1.33	2.14E-06	6.51	1.95E-08	0.06	4.66E-08	0.25	6	CHCl3 BDCM DBCM CHBr3 TTHM BrTHM
356.2409@7.4433494	3.30E-08	0.07	2.78E-08	0.33	1.51E-07	1.4	1.42E-06	6.84	2.43E-08	0.06	3.71E-08	0.26	6	CHCl3 BDCM DBCM CHBr3 TTHM BrTHM
282.2555@7.504335	1.78E-06	0.07	8.92E-07	0.32	2.82E-06	1.39	7.61E-06	6.96	1.26E-06	0.06	1.04E-06	0.25	6	CHCl3 BDCM DBCM CHBr3 TTHM BrTHM
618.4318@7.504522	1.71E-07	0.07	3.22E-07	0.3	1.24E-06	1.28	2.96E-06	6.5	1.55E-07	0.05	3.90E-07	0.24	6	CHCl3 BDCM DBCM CHBr3 TTHM BrTHM
602.4565@7.504135	3.35E-07	0.07	3.02E-07	0.33	1.36E-06	1.4	7.39E-06	6.89	2.64E-07	0.06	3.90E-07	0.26	6	CHCl3 BDCM DBCM CHBr3 TTHM BrTHM
354.2538@7.512989	6.63E-07	0.07	1.04E-07	0.35	2.32E-07	1.52	1.17E-06	7.55	3.50E-07	0.06	1.12E-07	0.27	6	CHCl3 BDCM DBCM CHBr3 TTHM BrTHM
338.2672@7.759727	1.70E-07	0.07	1.11E-07	0.3	4.86E-07	1.28	2.50E-06	6.33	1.22E-07	0.05	1.40E-07	0.23	6	CHCl3 BDCM DBCM CHBr3 TTHM BrTHM
433.2607@7.7598405	6.17E-07	0.06	1.80E-07	0.3	3.69E-07	1.3	1.01E-06	6.54	3.69E-07	0.05	1.87E-07	0.24	6	CHCl3 BDCM DBCM CHBr3 TTHM BrTHM
574.1899@7.7600746	2.37E-06	0.06	5.82E-07	0.3	5.42E-07	1.32	1.10E-06	6.73	1.36E-06	0.05	5.15E-07	0.23	6	CHCl3 BDCM DBCM CHBr3 TTHM BrTHM
370.2934@7.7597647	6.10E-07	0.07	2.04E-07	0.3	4.12E-07	1.33	1.01E-06	6.73	3.73E-07	0.05	2.12E-07	0.24	6	CHCl3 BDCM DBCM CHBr3 TTHM BrTHM
372.2175@7.7601714	1.42E-06	0.07	6.06E-07	0.31	1.47E-06	1.33	4.00E-06	6.65	9.47E-07	0.05	6.68E-07	0.24	6	CHCl3 BDCM DBCM CHBr3 TTHM BrTHM
401.2353@7.7597713	6.74E-07	0.07	2.75E-07	0.3	7.13E-07	1.32	2.71E-06	6.55	4.41E-07	0.05	3.07E-07	0.24	6	CHCl3 BDCM DBCM CHBr3 TTHM BrTHM
402.3193@7.759077	7.20E-06	0.06	2.67E-06	0.27	3.79E-06	1.21	6.55E-06	6.17	4.71E-06	0.05	2.60E-06	0.22	6	CHCl3 BDCM DBCM CHBr3 TTHM BrTHM
416.2993@7.7595434	6.52E-07	0.07	1.54E-07	0.32	2.78E-07	1.41	7.68E-07	7.11	3.70E-07	0.06	1.56E-07	0.26	6	CHCl3 BDCM DBCM CHBr3 TTHM BrTHM
370.2171@7.7601023	1.50E-06	0.07	8.73E-07	0.3	1.94E-06	1.29	6.04E-06	6.46	1.07E-06	0.05	9.51E-07	0.24	6	CHCl3 BDCM DBCM CHBr3 TTHM BrTHM
384.2723@7.7603097	1.72E-07	0.07	6.78E-08	0.32	2.12E-07	1.39	8.85E-07	6.93	1.08E-07	0.06	7.78E-08	0.25	6	CHCl3 BDCM DBCM CHBr3 TTHM BrTHM
384.1969@7.760461	3.08E-07	0.06	2.31E-07	0.29	7.18E-07	1.25	2.58E-06	6.22	2.28E-07	0.05	2.68E-07	0.23	6	CHCl3 BDCM DBCM CHBr3 TTHM BrTHM
368.2234@7.760178	7.01E-07	0.07	4.08E-07	0.31	1.05E-06	1.34	3.48E-06	6.69	4.95E-07	0.05	4.56E-07	0.24	6	CHCl3 BDCM DBCM CHBr3 TTHM BrTHM
379.2172@7.760255	2.78E-07	0.07	1.82E-07	0.32	4.59E-07	1.39	2.07E-06	6.86	1.97E-07	0.06	2.03E-07	0.25	6	CHCl3 BDCM DBCM CHBr3 TTHM BrTHM
443.246@7.7603326	6.33E-07	0.07	3.73E-08	0.32	2.20E-08	1.43	2.42E-08	7.42	2.55E-07	0.05	2.82E-08	0.25	6	CHCl3 BDCM DBCM CHBr3 TTHM BrTHM
364.2821@7.817511	1.82E-07	0.07	9.37E-08	0.34	4.20E-07	1.44	2.16E-06	7.14	1.24E-07	0.06	1.18E-07	0.26	6	CHCl3 BDCM DBCM CHBr3 TTHM BrTHM
364.2098@7.8170333	4.53E-08	0.08	1.94E-08	0.35	6.81E-08	1.51	2.39E-07	7.61	2.80E-08	0.06	2.23E-08	0.28	6	CHCl3 BDCM DBCM CHBr3 TTHM BrTHM
723.5196@8.9707155	2.05E-06	-0.01	2.26E-07	-0.04	1.70E-07	-0.17	3.02E-07	-0.85	9.81E-07	-0.01	1.87E-07	-0.03	6	CHCl3 BDCM DBCM CHBr3 TTHM BrTHM
205.9728@1.8373216	1.07E-14	0.09	6.87E-16	0.39	3.91E-14	1.69	9.17E-12	8.29	2.89E-15	0.07	1.36E-15	0.31	6	CHCl3 BDCM DBCM CHBr3 TTHM BrTHM
480.239@6.820197	3.65E-07	-0.04	7.39E-07	-0.2	1.49E-06	-0.86	1.64E-06	-4.45	3.29E-07	-0.04	7.58E-07	-0.16	6	CHCl3 BDCM DBCM CHBr3 TTHM BrTHM
492.2188@6.826548	5.16E-07	-0.04	5.11E-07	-0.18	6.60E-07	-0.8	1.69E-06	-4.04	3.92E-07	-0.03	4.88E-07	-0.14	6	CHCl3 BDCM DBCM CHBr3 TTHM BrTHM
589.3136@6.9212685	3.51E-08	0.03	1.45E-08	0.13	4.25E-08	0.59	6.30E-07	2.85	2.14E-08	0.02	1.70E-08	0.11	6	CHCl3 BDCM DBCM CHBr3 TTHM BrTHM
310.147@7.238965	2.61E-06	0.07	5.72E-07	0.32	1.57E-06	1.4	7.26E-06	6.87	1.55E-06	0.06	6.53E-07	0.26	6	CHCl3 BDCM DBCM CHBr3 TTHM BrTHM
374.288@7.443729	7.78E-10	0.08	7.31E-10	0.36	7.30E-09	1.55	8.44E-08	7.63	5.38E-10	0.06	1.07E-09	0.28	6	CHCl3 BDCM DBCM CHBr3 TTHM BrTHM
376.2121@7.442867	4.06E-08	0.08	3.86E-08	0.34	2.43E-07	1.43	2.26E-06	6.96	3.12E-08	0.06	5.35E-08	0.26	6	CHCl3 BDCM DBCM CHBr3 TTHM BrTHM
373.2046@7.443024	6.55E-08	0.07	9.78E-08	0.31	6.60E-07	1.32	6.08E-06	6.39	5.71E-08	0.06	1.40E-07	0.24	6	CHCl3 BDCM DBCM CHBr3 TTHM BrTHM
451.2712@7.759834	3.66E-06	0.06	1.09E-06	0.29	2.02E-06	1.26	4.84E-06	6.34	2.30E-06	0.05	1.13E-06	0.23	6	CHCl3 BDCM DBCM CHBr3 TTHM BrTHM
258.9906@1.3558017	1.36E-07	0.07	7.08E-08	0.32	4.39E-07	1.35	5.50E-06	6.27	9.43E-08	0.06	1.01E-07	0.25	6	CHCl3 BDCM DBCM CHBr3 TTHM BrTHM
256.9669@1.3459996	5.71E-12	0.08	3.33E-12	0.34	2.55E-10	1.44	1.25E-07	6.63	3.45E-12	0.06	8.89E-12	0.27	6	CHCl3 BDCM DBCM CHBr3 TTHM BrTHM
230.9969@1.8362806	4.24E-09	0.08	2.30E-10	0.35	4.65E-10	1.57	3.46E-09	7.9	1.53E-09	0.06	2.26E-10	0.28	6	CHCl3 BDCM DBCM CHBr3 TTHM BrTHM
207.9678@1.8371929	6.85E-07	0.06	5.16E-08	0.29	1.20E-07	1.29	9.09E-07	6.35	3.13E-07	0.05	5.68E-08	0.23	6	CHCl3 BDCM DBCM CHBr3 TTHM BrTHM
193.0147@1.8345736	7.96E-12	0.08	2.04E-13	0.38	5.26E-12	1.64	5.16E-10	8.01	2.25E-12	0.07	3.60E-13	0.3	6	CHCl3 BDCM DBCM CHBr3 TTHM BrTHM
413.1779@7.2739706	1.37E-06	0.05	1.83E-07	0.25	2.21E-07	1.09	5.82E-07	5.55	6.91E-07	0.04	1.70E-07	0.2	6	CHCl3 BDCM DBCM CHBr3 TTHM BrTHM
502.1657@7.4449286	2.16E-06	0.04	9.07E-07	0.16	8.29E-07	0.74	5.97E-07	3.89	1.38E-06	0.03	7.75E-07	0.13	6	CHCl3 BDCM DBCM CHBr3 TTHM BrTHM
373.2069@7.4430933	8.12E-08	0.07	1.02E-07	0.31	6.46E-07	1.32	6.06E-06	6.39	6.83E-08	0.06	1.44E-07	0.24	6	CHCl3 BDCM DBCM CHBr3 TTHM BrTHM
346.216@7.5908046	4.98E-08	0.07	8.93E-09	0.32	1.96E-08	1.4	5.48E-08	7.13	2.49E-08	0.05	9.07E-09	0.25	6	CHCl3 BDCM DBCM CHBr3 TTHM BrTHM
530.198@7.7611513	3.62E-08	0.05	4.18E-08	0.22	8.53E-08	0.96	1.57E-07	4.91	2.70E-08	0.04	4.20E-08	0.17	6	CHCl3 BDCM DBCM CHBr3 TTHM BrTHM
234.9896@1.3606412	2.68E-07	0.07	9.28E-08	0.32	4.50E-07	1.37	6.91E-06	6.53	1.71E-07	0.06	1.26E-07	0.25	6	CHCl3 BDCM DBCM CHBr3 TTHM BrTHM
493.1525@5.2638583	5.29E-09	-0.07	5.50E-09	-0.32	6.90E-08	-1.37	3.34E-06	-6.41	4.11E-09	-0.06	9.47E-09	-0.25	6	CHCl3 BDCM DBCM CHBr3 TTHM BrTHM
522.2935@7.043347	1.37E-06	-0.04	3.10E-07	-0.17	3.75E-07	-0.74	1.85E-06	-3.64	7.73E-07	-0.03	2.96E-07	-0.13	6	CHCl3 BDCM DBCM CHBr3 TTHM BrTHM
316.2963@7.4482174	8.67E-08	0.07	9.26E-09	0.33	6.54E-08	1.39	1.62E-06	6.62	4.27E-08	0.06	1.35E-08	0.26	6	CHCl3 BDCM DBCM CHBr3 TTHM BrTHM

383.205@7.7607985	3.13E-07	0.07	2.28E-07	0.32	7.72E-07	1.37	1.42E-06	6.97	2.29E-07	0.06	2.62E-07	0.25	6	CHCl3 BDCM DBCM CHBr3 TTHM BrTHM
289.1104@6.511069	3.01E-12	0.08	4.28E-10	0.35	4.42E-08	1.44	7.25E-06	6.48	7.56E-12	0.07	1.30E-09	0.28	6	CHCl3 BDCM DBCM CHBr3 TTHM BrTHM
124.0124@1.3471073	1.42E-09	0.07	1.20E-09	0.32	1.68E-08	1.35	5.37E-07	6.34	1.01E-09	0.06	2.04E-09	0.25	6	CHCl3 BDCM DBCM CHBr3 TTHM BrTHM
374.1704@4.1407986	1.05E-14	0.08	3.07E-14	0.34	5.28E-12	1.44	5.09E-09	6.67	7.40E-15	0.06	9.05E-14	0.27	6	CHCl3 BDCM DBCM CHBr3 TTHM BrTHM
448.2077@4.154416	7.48E-14	0.07	3.14E-12	0.31	9.86E-10	1.26	5.27E-07	5.66	1.21E-13	0.06	1.20E-11	0.24	6	CHCl3 BDCM DBCM CHBr3 TTHM BrTHM
540.2141@4.879549	8.34E-16	0.09	6.27E-15	0.4	2.80E-12	1.67	4.54E-09	7.76	7.15E-16	0.07	2.41E-14	0.31	6	CHCl3 BDCM DBCM CHBr3 TTHM BrTHM
385.2115@7.4435143	6.68E-07	0.06	8.96E-07	0.26	2.21E-06	1.14	6.39E-06	5.72	5.69E-07	0.05	9.99E-07	0.21	6	CHCl3 BDCM DBCM CHBr3 TTHM BrTHM
394.3589@9.49973	1.07E-08	-0.05	1.63E-08	-0.22	6.96E-08	-0.96	3.59E-07	-4.8	8.57E-09	-0.04	1.99E-08	-0.18	6	CHCl3 BDCM DBCM CHBr3 TTHM BrTHM
408.3749@9.502809	1.69E-15	-0.06	4.05E-14	-0.24	8.21E-13	-1.04	1.04E-11	-5.27	1.58E-15	-0.04	5.89E-14	-0.19	6	CHCl3 BDCM DBCM CHBr3 TTHM BrTHM
549.3239@7.2768703	5.00E-06	-0.05	5.34E-07	-0.24	6.89E-07	-1.08	2.45E-06	-5.42	2.51E-06	-0.04	5.16E-07	-0.19	6	CHCl3 BDCM DBCM CHBr3 TTHM BrTHM
687.388@7.443929	1.82E-08	0.07	3.27E-09	0.31	9.60E-09	1.36	8.81E-08	6.76	9.06E-09	0.05	3.66E-09	0.25	6	CHCl3 BDCM DBCM CHBr3 TTHM BrTHM
278.0627@1.8338702	9.38E-09	0.07	1.54E-09	0.34	1.25E-08	1.45	1.74E-07	7.11	4.72E-09	0.06	2.21E-09	0.27	6	CHCl3 BDCM DBCM CHBr3 TTHM BrTHM
270.0341@1.8323116	1.20E-06	0.06	1.03E-07	0.29	8.54E-08	1.31	6.91E-08	6.81	5.37E-07	0.05	8.44E-08	0.23	6	CHCl3 BDCM DBCM CHBr3 TTHM BrTHM
478.2383@6.820434	1.18E-06	-0.04	2.14E-06	-0.2	4.36E-06	-0.85	4.91E-06	-4.42	1.07E-06	-0.04	2.23E-06	-0.15	6	CHCl3 BDCM DBCM CHBr3 TTHM BrTHM
362.1766@7.42454	1.83E-07	0.07	9.00E-08	0.31	3.56E-07	1.35	1.19E-06	6.81	1.22E-07	0.06	1.09E-07	0.25	6	CHCl3 BDCM DBCM CHBr3 TTHM BrTHM
356.2775@7.759538	1.26E-08	0.07	6.44E-10	0.35	8.10E-10	1.57	1.33E-08	7.74	4.59E-09	0.06	5.97E-10	0.28	6	CHCl3 BDCM DBCM CHBr3 TTHM BrTHM
724.3821@7.443623	4.89E-09	0.07	2.48E-08	0.31	1.84E-07	1.34	1.02E-06	6.65	5.30E-09	0.06	3.48E-08	0.25	6	CHCl3 BDCM DBCM CHBr3 TTHM BrTHM
342.186@7.443287	7.47E-08	0.07	1.22E-07	0.32	7.77E-07	1.36	7.40E-06	6.59	6.66E-08	0.06	1.73E-07	0.25	6	CHCl3 BDCM DBCM CHBr3 TTHM BrTHM
398.1707@5.2510157	1.31E-12	0.08	2.12E-12	0.36	1.21E-10	1.54	1.41E-08	7.41	9.24E-13	0.06	4.87E-12	0.29	6	CHCl3 BDCM DBCM CHBr3 TTHM BrTHM
396.1522@4.144505	5.08E-17	0.08	1.81E-16	0.35	2.54E-13	1.44	3.79E-09	6.46	3.41E-17	0.06	9.60E-16	0.27	6	CHCl3 BDCM DBCM CHBr3 TTHM BrTHM
369.2754@7.5048666	1.87E-07	0.07	1.33E-07	0.32	6.33E-07	1.39	5.89E-06	6.71	1.38E-07	0.06	1.75E-07	0.26	6	CHCl3 BDCM DBCM CHBr3 TTHM BrTHM
220.0238@1.8354434	1.59E-11	0.08	7.45E-13	0.35	1.56E-11	1.52	9.08E-10	7.47	5.16E-12	0.06	1.25E-12	0.28	6	CHCl3 BDCM DBCM CHBr3 TTHM BrTHM
206.996@1.8341223	2.56E-09	0.07	3.37E-11	0.35	6.35E-11	1.56	4.96E-10	7.88	6.83E-10	0.06	3.16E-11	0.28	6	CHCl3 BDCM DBCM CHBr3 TTHM BrTHM
769.958@7.041837	8.65E-06	-0.04	3.09E-06	-0.18	3.04E-06	-0.79	3.00E-06	-4.09	5.53E-06	-0.03	2.73E-06	-0.14	5	BDCM DBCM CHBr3 TTHM BrTHM
289.1441@7.2778654	1.00E-05	-0.05	1.88E-06	-0.24	1.51E-06	-1.09	1.70E-06	-5.66	5.58E-06	-0.04	1.59E-06	-0.19	5	BDCM DBCM CHBr3 TTHM BrTHM
218.9947@1.351686	2.66E-07	0.07	1.10E-07	0.3	7.00E-07	1.25	2.37E-05	5.53	1.81E-07	0.05	1.65E-07	0.23	5	CHCl3 BDCM DBCM TTHM BrTHM
200.9487@1.347976	4.68E-06	0.06	1.23E-06	0.28	5.51E-06	1.17	1.12E-04	5.24	3.05E-06	0.05	1.72E-06	0.22	5	CHCl3 BDCM DBCM TTHM BrTHM
86.037@1.359864	1.91E-07	0.07	1.16E-07	0.32	8.65E-07	1.33	1.45E-05	6.03	1.40E-07	0.06	1.73E-07	0.25	5	CHCl3 BDCM DBCM TTHM BrTHM
261.0053@1.5883832	5.87E-06	0.06	1.41E-06	0.27	3.69E-06	1.18	2.94E-05	5.47	3.64E-06	0.05	1.67E-06	0.22	5	CHCl3 BDCM DBCM TTHM BrTHM
202.9642@1.5914642	4.14E-07	0.07	1.19E-07	0.3	7.65E-07	1.27	1.66E-05	5.76	2.62E-07	0.05	1.74E-07	0.24	5	CHCl3 BDCM DBCM TTHM BrTHM
261.0054@1.6265397	6.01E-07	0.07	5.14E-07	0.3	2.56E-06	1.28	8.71E-05	5.56	4.86E-07	0.05	7.40E-07	0.24	5	CHCl3 BDCM DBCM TTHM BrTHM
126.0292@1.5888929	3.59E-07	0.07	1.81E-07	0.31	1.31E-06	1.31	2.43E-05	5.98	2.57E-07	0.05	2.70E-07	0.24	5	CHCl3 BDCM DBCM TTHM BrTHM
234.005@1.8347428	5.56E-06	0.05	8.92E-07	0.26	1.35E-06	1.14	8.90E-06	5.57	3.08E-06	0.04	9.19E-07	0.2	5	CHCl3 BDCM DBCM TTHM BrTHM
169.0367@2.3033211	5.38E-06	-0.05	1.43E-06	-0.24	3.11E-06	-1.03	1.05E-05	-4.71	3.38E-06	-0.04	1.60E-06	-0.19	5	CHCl3 BDCM DBCM TTHM BrTHM
187.0637@2.5992222	2.74E-07	-0.05	5.94E-07	-0.23	2.57E-06	-0.98	9.42E-06	-4.89	2.64E-07	-0.04	7.65E-07	-0.18	5	CHCl3 BDCM DBCM TTHM BrTHM
117.0577@2.598943	1.23E-06	-0.05	1.45E-06	-0.23	5.30E-06	-0.98	2.33E-05	-4.81	1.06E-06	-0.04	1.83E-06	-0.18	5	CHCl3 BDCM DBCM TTHM BrTHM
559.1793@2.6203928	1.11E-07	-0.04	4.97E-07	-0.17	2.82E-06	-0.73	1.50E-05	-3.59	1.27E-07	-0.03	6.89E-07	-0.14	5	CHCl3 BDCM DBCM TTHM BrTHM
225.0637@2.742921	7.51E-09	-0.07	2.54E-08	-0.31	3.85E-07	-1.29	2.74E-05	-5.41	7.86E-09	-0.06	4.66E-08	-0.24	5	CHCl3 BDCM DBCM TTHM BrTHM
260.1365@3.0406637	1.25E-07	-0.05	2.07E-07	-0.21	9.83E-07	-0.88	3.19E-05	-4.03	1.13E-07	-0.04	2.92E-07	-0.16	5	CHCl3 BDCM DBCM TTHM BrTHM
562.1946@4.8801045	3.03E-11	0.08	5.58E-10	0.35	6.43E-08	1.43	1.26E-05	6.3	4.71E-11	0.06	1.73E-09	0.27	5	CHCl3 BDCM DBCM TTHM BrTHM
273.1755@5.3126297	6.22E-07	0.05	5.91E-08	0.24	2.13E-07	1.05	9.60E-06	4.83	3.08E-07	0.04	7.79E-08	0.19	5	CHCl3 BDCM DBCM TTHM BrTHM
494.1572@5.263294	9.45E-07	-0.06	2.58E-07	-0.29	1.12E-06	-1.23	1.47E-05	-5.86	5.92E-07	-0.05	3.44E-07	-0.23	5	CHCl3 BDCM DBCM TTHM BrTHM
304.0978@6.5103226	3.14E-09	0.08	6.99E-08	0.34	1.57E-06	1.38	5.18E-05	6.3	5.39E-09	0.06	1.42E-07	0.26	5	CHCl3 BDCM DBCM TTHM BrTHM
290.1171@6.510924	1.57E-10	0.08	4.92E-09	0.33	2.23E-07	1.35	1.75E-05	6.1	2.82E-10	0.06	1.21E-08	0.26	5	CHCl3 BDCM DBCM TTHM BrTHM
272.1503@6.5114565	4.27E-10	0.08	1.12E-08	0.33	4.45E-07	1.34	1.88E-05	6.11	7.42E-10	0.06	2.58E-08	0.26	5	CHCl3 BDCM DBCM TTHM BrTHM
374.2812@6.816042	4.27E-07	-0.05	1.30E-06	-0.2	3.71E-06	-0.88	8.74E-06	-4.41	4.37E-07	-0.04	1.49E-06	-0.16	5	CHCl3 BDCM DBCM TTHM BrTHM
522.292@7.043714	2.20E-06	-0.03	1.73E-06	-0.15	4.46E-06	-0.66	1.37E-05	-3.29	1.71E-06	-0.03	1.97E-06	-0.12	5	CHCl3 BDCM DBCM TTHM BrTHM
328.1338@7.174319	1.68E-06	0.06	1.29E-06	0.28	4.28E-06	1.19	3.47E-05	5.73	1.32E-06	0.05	1.61E-06	0.22	5	CHCl3 BDCM DBCM TTHM BrTHM
332.1435@7.1832304	7.42E-06	0.06	3.10E-06	0.28	7.61E-06	1.22	4.13E-05	5.92	5.18E-06	0.05	3.59E-06	0.22	5	CHCl3 BDCM DBCM TTHM BrTHM
334.2354@7.326612	1.82E-06	0.07	1.07E-06	0.3	4.38E-06	1.28	2.40E-05	6.26	1.36E-06	0.05	1.38E-06	0.24	5	CHCl3 BDCM DBCM TTHM BrTHM
348.2146@7.320005	1.01E-06	0.07	9.61E-07	0.3	3.33E-06	1.27	1.43E-05	6.25	8.21E-07	0.05	1.18E-06	0.23	5	CHCl3 BDCM DBCM TTHM BrTHM
318.1876@7.32732	3.49E-06	0.06	2.11E-06	0.29	7.43E-06	1.23	3.08E-05	6.07	2.63E-06	0.05	2.61E-06	0.23	5	CHCl3 BDCM DBCM TTHM BrTHM
374.2105@7.443445	1.19E-07	0.07	1.87E-07	0.31	1.16E-06	1.33	9.18E-06	6.43	1.06E-07	0.06	2.62E-07	0.25	5	CHCl3 BDCM DBCM TTHM BrTHM
388.2674@7.443495	6.27E-06	0.06	3.21E-06	0.29	5.71E-06	1.27	1.36E-05	6.36	4.48E-06	0.05	3.36E-06	0.23	5	CHCl3 BDCM DBCM TTHM BrTHM
356.1659@7.444197	1.64E-06	0.06	1.15E-06	0.26	5.78E-06	1.1	5.05E-05	5.19	1.29E-06	0.05	1.58E-06	0.2	5	CHCl3 BDCM DBCM TTHM BrTHM
351.1855@7.4437146	6.28E-07	0.07	7.13E-07	0.29	2.91E-06	1.24	1.38E-05	6.12	5.30E-07	0.05	9.07E-07	0.23	5	CHCl3 BDCM DBCM TTHM BrTHM
383.2113@7.4437904	5.11E-06	0.06	4.23E-06	0.26	7.36E-06	1.12	1.42E-05	5.72	4.03E-06	0.05	4.39E-06	0.2	5	CHCl3 BDCM DBCM TTHM BrTHM
441.2311@7.504525	1.67E-06	0.07	1.28E-06	0.32	4.56E-06	1.35	2.00E-05	6.64	1.31E-06	0.06	1.59E-06	0.25	5	CHCl3 BDCM DBCM TTHM BrTHM
390.2726@7.504331	1.75E-07	0.07	3.26E-07	0.3	1.63E-06	1.3	8.70E-06	6.37	1.62E-07	0.05	4.33E-07	0.24	5	CHCl3 BDCM DBCM TTHM BrTHM

368.2775@7.500682	1.35E-06	0.07	1.03E-06	0.31	3.12E-06	1.32	9.08E-06	6.6	1.04E-06	0.05	1.20E-06	0.24	5	CHCl3 BDCM DBCM TTHM BrTHM
363.1983@7.498433	4.20E-06	0.07	1.58E-06	0.32	4.52E-06	1.37	2.52E-05	6.61	2.85E-06	0.05	1.87E-06	0.25	5	CHCl3 BDCM DBCM TTHM BrTHM
336.2516@7.504455	1.00E-06	0.07	8.05E-07	0.31	3.25E-06	1.33	1.50E-05	6.54	7.88E-07	0.05	1.02E-06	0.25	5	CHCl3 BDCM DBCM TTHM BrTHM
336.1749@7.5043306	1.55E-06	0.07	1.53E-06	0.3	6.31E-06	1.3	3.05E-05	6.33	1.30E-06	0.05	1.97E-06	0.24	5	CHCl3 BDCM DBCM TTHM BrTHM
320.2027@7.5044994	5.29E-07	0.07	4.24E-07	0.31	1.80E-06	1.3	8.15E-06	6.45	4.08E-07	0.05	5.38E-07	0.24	5	CHCl3 BDCM DBCM TTHM BrTHM
382.2554@7.502282	7.80E-07	0.07	7.10E-07	0.29	3.02E-06	1.26	1.42E-05	6.17	6.30E-07	0.05	9.09E-07	0.23	5	CHCl3 BDCM DBCM TTHM BrTHM
572.1735@7.5062647	8.94E-07	0.07	1.12E-06	0.31	5.52E-06	1.29	2.62E-05	6.35	7.85E-07	0.05	1.50E-06	0.24	5	CHCl3 BDCM DBCM TTHM BrTHM
404.243@7.7596955	3.43E-06	0.06	1.33E-06	0.3	3.21E-06	1.28	1.01E-05	6.36	2.30E-06	0.05	1.49E-06	0.23	5	CHCl3 BDCM DBCM TTHM BrTHM
381.7682@9.035833	1.29E-06	0.02	5.97E-07	0.08	3.66E-06	0.32	1.64E-04	1.43	9.43E-07	0.01	9.24E-07	0.06	5	CHCl3 BDCM DBCM TTHM BrTHM
355.2716@6.811236	6.65E-07	-0.05	2.24E-06	-0.2	5.78E-06	-0.88	9.81E-06	-4.45	7.00E-07	-0.04	2.50E-06	-0.16	5	CHCl3 BDCM DBCM TTHM BrTHM
446.2109@6.805361	9.32E-07	-0.04	1.54E-06	-0.18	4.61E-06	-0.78	1.23E-05	-3.92	8.45E-07	-0.03	1.80E-06	-0.14	5	CHCl3 BDCM DBCM TTHM BrTHM
364.192@7.32695	3.53E-06	0.07	1.85E-06	0.3	5.63E-06	1.3	2.82E-05	6.35	2.57E-06	0.05	2.23E-06	0.24	5	CHCl3 BDCM DBCM TTHM BrTHM
104.0473@1.3595326	9.91E-08	0.07	1.10E-07	0.32	8.42E-07	1.36	2.03E-05	6.24	8.23E-08	0.06	1.68E-07	0.25	5	CHCl3 BDCM DBCM TTHM BrTHM
205.9733@1.6010213	1.99E-06	0.07	6.73E-07	0.3	2.93E-06	1.29	1.10E-05	6.41	1.32E-06	0.05	8.60E-07	0.24	5	CHCl3 BDCM DBCM TTHM BrTHM
302.11@6.5075555	1.47E-09	0.08	2.94E-08	0.32	4.49E-07	1.35	8.72E-06	6.39	2.31E-09	0.06	5.23E-08	0.25	5	CHCl3 BDCM DBCM TTHM BrTHM
340.1729@7.238439	3.60E-06	0.07	1.52E-06	0.31	4.80E-06	1.35	2.02E-05	6.63	2.50E-06	0.05	1.81E-06	0.25	5	CHCl3 BDCM DBCM TTHM BrTHM
349.1728@7.238255	2.45E-06	0.07	1.33E-06	0.3	4.50E-06	1.3	1.66E-05	6.42	1.79E-06	0.05	1.60E-06	0.24	5	CHCl3 BDCM DBCM TTHM BrTHM
395.2249@7.8182287	1.30E-06	0.07	1.11E-06	0.3	5.50E-06	1.28	3.66E-05	6.15	1.06E-06	0.05	1.50E-06	0.24	5	CHCl3 BDCM DBCM TTHM BrTHM
335.1778@7.5041614	2.18E-06	0.07	1.78E-06	0.3	7.65E-06	1.28	3.10E-05	6.26	1.76E-06	0.05	2.30E-06	0.24	5	CHCl3 BDCM DBCM TTHM BrTHM
342.2257@7.817047	2.32E-06	0.07	1.46E-06	0.3	4.80E-06	1.3	1.51E-05	6.49	1.74E-06	0.05	1.75E-06	0.24	5	CHCl3 BDCM DBCM TTHM BrTHM
292.1182@6.5112	2.87E-11	0.08	1.26E-09	0.34	7.89E-08	1.41	9.46E-06	6.36	5.45E-11	0.06	3.36E-09	0.27	5	CHCl3 BDCM DBCM TTHM BrTHM
310.1454@7.237161	2.10E-06	0.07	7.19E-07	0.32	2.20E-06	1.38	9.71E-06	6.8	1.38E-06	0.06	8.40E-07	0.25	5	CHCl3 BDCM DBCM TTHM BrTHM
367.1945@7.504806	1.63E-06	0.07	1.35E-06	0.3	5.18E-06	1.27	2.24E-05	6.23	1.31E-06	0.05	1.69E-06	0.23	5	CHCl3 BDCM DBCM TTHM BrTHM
356.2711@6.815725	7.57E-07	-0.05	2.23E-06	-0.2	6.15E-06	-0.86	1.39E-05	-4.31	7.79E-07	-0.04	2.55E-06	-0.16	5	CHCl3 BDCM DBCM TTHM BrTHM
523.2926@7.0411916	2.45E-06	-0.04	1.98E-06	-0.16	4.32E-06	-0.72	1.91E-05	-3.52	1.91E-06	-0.03	2.20E-06	-0.13	5	CHCl3 BDCM DBCM TTHM BrTHM
726.3785@7.4434447	1.30E-07	0.07	2.50E-07	0.29	1.48E-06	1.23	1.20E-05	5.94	1.22E-07	0.05	3.51E-07	0.23	5	CHCl3 BDCM DBCM TTHM BrTHM
126.029@1.5888929	3.23E-07	0.07	1.52E-07	0.31	1.10E-06	1.32	1.90E-05	6.04	2.27E-07	0.05	2.26E-07	0.25	5	CHCl3 BDCM DBCM TTHM BrTHM
360.1945@7.590344	3.41E-06	0.06	1.72E-06	0.29	5.11E-06	1.24	2.50E-05	6.05	2.46E-06	0.05	2.06E-06	0.23	5	CHCl3 BDCM DBCM TTHM BrTHM
619.4304@7.5045266	4.05E-07	0.07	5.60E-07	0.3	2.41E-06	1.27	9.25E-06	6.33	3.53E-07	0.05	7.13E-07	0.23	5	CHCl3 BDCM DBCM TTHM BrTHM
205.973@1.6046426	1.42E-06	0.07	1.03E-06	0.31	4.71E-06	1.32	3.09E-05	6.39	1.11E-06	0.05	1.37E-06	0.24	5	CHCl3 BDCM DBCM TTHM BrTHM
358.2021@7.592699	1.59E-06	0.06	7.44E-07	0.28	3.25E-06	1.18	2.46E-05	5.68	1.13E-06	0.05	9.79E-07	0.22	5	CHCl3 BDCM DBCM TTHM BrTHM
144.0239@1.9036608	6.31E-06	-0.02	2.87E-06	-0.1	3.17E-06	-0.44	4.36E-05	-2.09	4.34E-06	-0.02	2.90E-06	-0.08	5	CHCl3 BDCM DBCM TTHM BrTHM
86.0371@1.5957161	4.11E-06	0.07	9.94E-07	0.32	3.34E-06	1.36	3.20E-05	6.49	2.56E-06	0.05	1.25E-06	0.25	5	CHCl3 BDCM DBCM TTHM BrTHM
344.1833@7.4433827	1.29E-07	0.07	2.08E-07	0.32	1.36E-06	1.34	1.22E-05	6.45	1.17E-07	0.06	2.98E-07	0.25	5	CHCl3 BDCM DBCM TTHM BrTHM
382.1594@7.2746925	3.70E-05	0.05	2.34E-06	0.24	8.51E-07	1.11	1.98E-07	6.03	1.65E-05	0.04	1.61E-06	0.19	4	BDCM DBCM CHBr3 BrTHM
345.2688@7.27435	2.80E-05	0.05	3.82E-06	0.25	3.66E-06	1.1	4.57E-06	5.66	1.51E-05	0.04	3.40E-06	0.2	4	BDCM DBCM CHBr3 BrTHM
338.2781@7.4471393	3.90E-05	0.06	6.03E-06	0.28	4.27E-06	1.28	3.87E-06	6.68	2.15E-05	0.05	5.02E-06	0.23	4	BDCM DBCM CHBr3 BrTHM
392.2207@7.591975	1.66E-05	0.06	2.54E-06	0.29	2.68E-06	1.27	4.02E-06	6.55	9.14E-06	0.05	2.32E-06	0.23	4	BDCM DBCM CHBr3 BrTHM
437.197@7.76373	3.97E-05	0.05	3.12E-06	0.23	1.45E-06	1.04	1.81E-06	5.36	1.90E-05	0.04	2.39E-06	0.18	4	BDCM DBCM CHBr3 BrTHM
577.2847@7.2765718	7.27E-05	-0.04	6.42E-07	-0.19	3.44E-07	-0.85	1.58E-06	-4.22	2.40E-05	-0.03	5.19E-07	-0.15	4	BDCM DBCM CHBr3 BrTHM
126.0289@1.3600142	1.22E-05	0.06	2.44E-06	0.3	5.86E-06	1.3	2.29E-05	6.31	7.37E-06	0.05	2.78E-06	0.24	4	BDCM DBCM TTHM BrTHM
317.183@2.8920581	1.15E-05	0.04	1.79E-06	0.21	2.70E-06	0.94	2.27E-05	4.49	6.46E-06	0.04	1.88E-06	0.17	4	BDCM DBCM TTHM BrTHM
362.1899@7.5909405	1.00E-05	0.06	3.13E-06	0.28	4.25E-06	1.24	9.16E-06	6.24	6.40E-06	0.05	3.05E-06	0.22	4	BDCM DBCM TTHM BrTHM
122.037@3.195136	6.49E-06	-0.03	3.25E-06	-0.13	8.64E-06	-0.56	5.04E-05	-2.7	4.70E-06	-0.02	3.82E-06	-0.1	4	CHCl3 BDCM TTHM BrTHM
260.0351@4.977189	3.07E-07	0.06	2.16E-06	0.26	2.17E-05	1.05	4.92E-04	4.65	4.26E-07	0.05	3.73E-06	0.2	4	CHCl3 BDCM TTHM BrTHM
228.0671@5.9507146	2.24E-08	0.07	3.57E-07	0.3	7.92E-06	1.21	4.87E-04	5.15	3.79E-08	0.06	7.71E-07	0.23	4	CHCl3 BDCM TTHM BrTHM
293.1046@5.952404	3.47E-08	0.07	8.05E-07	0.29	1.70E-05	1.17	3.56E-04	5.22	6.44E-08	0.06	1.62E-06	0.23	4	CHCl3 BDCM TTHM BrTHM
371.3037@6.0384707	5.92E-06	0.04	1.91E-06	0.19	7.95E-06	0.79	1.13E-04	3.65	4.02E-06	0.03	2.59E-06	0.15	4	CHCl3 BDCM TTHM BrTHM
305.1101@6.0596585	1.45E-07	-0.02	1.43E-06	-0.07	8.92E-06	-0.29	3.14E-05	-1.45	2.05E-07	-0.01	2.01E-06	-0.05	4	CHCl3 BDCM TTHM BrTHM
256.0985@6.511782	1.33E-07	0.07	3.47E-06	0.27	5.77E-05	1.09	1.67E-03	4.61	2.66E-07	0.05	6.97E-06	0.21	4	CHCl3 BDCM TTHM BrTHM
365.1792@7.3263917	6.79E-06	0.06	3.82E-06	0.28	1.12E-05	1.2	5.03E-05	5.86	5.10E-06	0.05	4.58E-06	0.22	4	CHCl3 BDCM TTHM BrTHM
368.2003@7.5055165	5.47E-06	0.06	3.29E-06	0.29	1.03E-05	1.25	4.01E-05	6.12	4.15E-06	0.05	3.98E-06	0.23	4	CHCl3 BDCM TTHM BrTHM
382.1812@7.5048337	1.65E-06	0.06	1.99E-06	0.26	8.58E-06	1.11	3.97E-05	5.45	1.46E-06	0.05	2.60E-06	0.21	4	CHCl3 BDCM TTHM BrTHM
416.2231@7.7597513	6.14E-06	0.06	5.33E-06	0.26	1.18E-05	1.11	3.17E-05	5.52	4.98E-06	0.05	5.90E-06	0.2	4	CHCl3 BDCM TTHM BrTHM
428.2176@5.9300275	2.14E-06	-0.02	4.56E-06	-0.08	1.38E-05	-0.36	5.26E-05	-1.77	2.12E-06	-0.02	5.51E-06	-0.07	4	CHCl3 BDCM TTHM BrTHM
410.2079@7.7719355	2.76E-06	0.07	2.54E-06	0.31	7.81E-06	1.33	4.84E-05	6.38	2.27E-06	0.05	3.09E-06	0.24	4	CHCl3 BDCM TTHM BrTHM
399.1425@2.6059198	2.05E-06	-0.03	5.15E-06	-0.13	2.47E-05	-0.56	1.48E-04	-2.66	2.17E-06	-0.02	7.03E-06	-0.1	4	CHCl3 BDCM TTHM BrTHM
260.0946@6.5130615	1.97E-09	0.08	1.32E-06	0.3	3.47E-05	1.2	8.03E-04	5.22	9.25E-09	0.06	2.89E-06	0.23	4	CHCl3 BDCM TTHM BrTHM
526.7818@6.951364	2.30E-06	0.06	6.69E-06	0.27	1.44E-05	1.16	6.01E-05	5.67	2.41E-06	0.05	7.48E-06	0.21	4	CHCl3 BDCM TTHM BrTHM

262.0846@5.95244	7.28E-08	0.07	3.01E-06	0.3	9.75E-05	1.14	6.53E-03	4.18	1.68E-07	0.06	6.95E-06	0.23	4	CHCl3 BDCM TTHM BrTHM
272.0719@6.5123043	4.53E-08	0.07	2.66E-06	0.3	4.30E-05	1.19	3.03E-04	5.59	1.08E-07	0.06	4.90E-06	0.23	4	CHCl3 BDCM TTHM BrTHM
265.1722@4.9786844	8.74E-07	0.06	2.56E-06	0.24	1.91E-05	1.01	1.12E-04	4.83	9.67E-07	0.04	3.87E-06	0.19	4	CHCl3 BDCM TTHM BrTHM
268.0982@6.4982543	3.58E-07	0.05	1.14E-06	0.24	1.66E-05	0.96	3.45E-04	4.29	4.17E-07	0.04	2.13E-06	0.18	4	CHCl3 BDCM TTHM BrTHM
415.2775@6.8242917	1.76E-06	-0.05	5.42E-06	-0.21	1.87E-05	-0.88	1.19E-04	-4.2	1.92E-06	-0.04	6.89E-06	-0.16	4	CHCl3 BDCM TTHM BrTHM
351.1721@7.238381	1.43E-05	0.06	1.71E-06	0.31	3.39E-06	1.36	1.30E-05	6.72	7.75E-06	0.05	1.84E-06	0.25	3	BDCM DBCM BrTHM
549.8224@7.2789006	1.80E-05	-0.03	5.08E-06	-0.13	7.52E-06	-0.56	1.30E-05	-2.83	1.14E-05	-0.02	5.08E-06	-0.1	3	BDCM DBCM BrTHM
360.1745@7.4212384	2.08E-05	0.06	4.53E-06	0.26	6.31E-06	1.17	9.34E-06	5.97	1.25E-05	0.04	4.41E-06	0.21	3	BDCM DBCM BrTHM
769.9554@7.043136	2.33E-05	-0.04	5.85E-06	-0.17	5.48E-06	-0.75	8.04E-06	-3.83	1.42E-05	-0.03	5.24E-06	-0.13	3	BDCM DBCM BrTHM
361.1801@7.3264565	9.74E-06	0.06	4.49E-06	0.28	1.25E-05	1.19	5.18E-05	5.82	7.03E-06	0.05	5.34E-06	0.22	3	BDCM TTHM BrTHM
264.9476@1.3186778	9.98E-06	0.06	4.38E-06	0.28	1.50E-05	1.21	1.83E-04	5.56	7.27E-06	0.05	5.71E-06	0.22	3	BDCM TTHM BrTHM
572.1891@7.760543	8.28E-06	0.06	6.85E-06	0.26	1.48E-05	1.14	3.32E-05	5.7	6.66E-06	0.05	7.53E-06	0.21	3	BDCM TTHM BrTHM
415.2113@7.44384	6.18E-06	0.07	7.35E-06	0.29	2.30E-05	1.24	1.16E-04	5.97	5.51E-06	0.05	9.10E-06	0.23	3	CHCl3 BDCM TTHM
342.1534@7.3242216	7.50E-06	0.06	5.81E-06	0.28	2.43E-05	1.17	1.48E-04	5.61	6.20E-06	0.05	7.74E-06	0.22	3	CHCl3 BDCM TTHM
244.0241@1.3557141	1.88E-06	0.06	6.75E-06	0.28	2.64E-05	1.16	1.08E-04	5.61	2.14E-06	0.05	8.69E-06	0.22	3	CHCl3 BDCM TTHM
414.1171@5.004537	1.42E-06	0.06	6.73E-06	0.26	5.26E-05	1.05	5.44E-04	4.81	1.83E-06	0.05	1.08E-05	0.2	3	CHCl3 BDCM TTHM
471.2317@3.850803	3.44E-07	-0.04	6.19E-06	-0.17	3.60E-05	-0.69	3.17E-04	-3.2	5.87E-07	-0.03	9.08E-06	-0.13	3	CHCl3 BDCM TTHM
262.0848@5.9511933	4.95E-07	0.07	4.68E-06	0.28	5.60E-05	1.11	1.15E-03	4.83	7.57E-07	0.05	8.46E-06	0.21	3	CHCl3 BDCM TTHM
550.8251@7.2791157	5.90E-05	-0.03	8.74E-06	-0.14	5.31E-06	-0.61	3.13E-06	-3.25	3.23E-05	-0.02	7.03E-06	-0.11	3	DBCM CHBr3 BrTHM
330.2495@7.5914774	1.47E-05	0.06	8.61E-06	0.28	6.49E-06	1.26	7.67E-06	6.54	1.05E-05	0.05	7.35E-06	0.22	3	DBCM CHBr3 BrTHM
158.0883@2.5990798	1.16E-05	-0.05	6.99E-06	-0.23	1.37E-05	-1.01	4.48E-05	-5.02	8.80E-06	-0.04	7.68E-06	-0.18	2	BDCM BrTHM
336.1587@6.8894496	1.43E-05	0.05	6.61E-06	0.24	1.46E-05	1.04	4.84E-05	5.16	1.03E-05	0.04	7.41E-06	0.19	2	BDCM BrTHM
787.9693@7.043957	1.11E-05	-0.04	7.05E-06	-0.19	9.54E-06	-0.83	1.44E-05	-4.23	8.21E-06	-0.03	6.88E-06	-0.15	2	BDCM BrTHM
346.1786@7.1721168	1.16E-05	0.06	4.03E-06	0.28	1.13E-05	1.19	4.18E-05	5.84	7.94E-06	0.05	4.75E-06	0.22	2	BDCM BrTHM
340.1692@7.2387867	1.27E-05	0.07	3.00E-06	0.31	8.00E-06	1.34	3.74E-05	6.52	7.99E-06	0.05	3.49E-06	0.25	2	BDCM BrTHM
614.4@7.326449	1.07E-05	0.06	5.30E-06	0.28	1.50E-05	1.2	6.97E-05	5.82	7.89E-06	0.05	6.35E-06	0.22	2	BDCM BrTHM
262.2297@7.3250537	1.39E-05	0.06	4.80E-06	0.26	1.16E-05	1.13	1.11E-04	5.33	9.49E-06	0.05	5.73E-06	0.21	2	BDCM BrTHM
278.9827@3.194258	1.36E-05	-0.03	5.12E-06	-0.13	8.24E-06	-0.59	3.04E-05	-2.9	9.19E-06	-0.02	5.32E-06	-0.11	2	BDCM BrTHM
204.0509@1.8361962	1.61E-05	0.05	5.86E-06	0.24	1.63E-05	1.05	4.91E-05	5.2	1.12E-05	0.04	6.89E-06	0.19	2	BDCM BrTHM
366.1817@7.3246593	1.22E-05	0.06	6.50E-06	0.29	1.76E-05	1.23	7.09E-05	6.01	9.12E-06	0.05	7.67E-06	0.23	2	BDCM BrTHM
370.2046@7.5038757	1.42E-05	0.06	5.14E-06	0.29	1.43E-05	1.26	6.11E-05	6.13	9.82E-06	0.05	6.10E-06	0.23	2	BDCM BrTHM
296.1039@2.0677712	1.28E-05	-0.04	5.68E-06	-0.2	1.14E-05	-0.86	9.80E-05	-4.06	9.14E-06	-0.03	6.48E-06	-0.16	2	BDCM BrTHM
334.1596@7.325757	9.46E-06	0.06	7.12E-06	0.28	2.30E-05	1.18	1.10E-04	5.69	7.69E-06	0.05	8.83E-06	0.22	2	BDCM TTHM
379.2036@7.505036	9.02E-06	0.06	6.66E-06	0.28	2.02E-05	1.2	6.24E-05	5.95	7.24E-06	0.05	7.98E-06	0.22	2	BDCM TTHM
278.1266@2.2892509	2.38E-06	-0.03	2.69E-05	-0.12	1.63E-04	-0.5	5.45E-04	-2.4	3.77E-06	-0.02	3.88E-05	-0.09	2	CHCl3 TTHM
461.0902@2.5998218	4.94E-06	-0.05	1.88E-05	-0.22	1.09E-04	-0.9	5.25E-04	-4.27	5.98E-06	-0.04	2.69E-05	-0.17	2	CHCl3 TTHM
303.0069@2.5987859	1.86E-06	-0.05	1.09E-05	-0.21	5.90E-05	-0.85	2.14E-04	-4.15	2.44E-06	-0.04	1.53E-05	-0.16	2	CHCl3 TTHM
250.0971@3.5423863	5.02E-06	-0.04	4.04E-05	-0.17	1.22E-04	-0.71	7.86E-04	-3.31	7.14E-06	-0.03	5.10E-05	-0.13	2	CHCl3 TTHM
470.7311@3.849371	2.12E-06	-0.04	2.04E-05	-0.17	9.21E-05	-0.72	6.77E-04	-3.31	3.17E-06	-0.03	2.83E-05	-0.13	2	CHCl3 TTHM
261.079@5.9514775	8.89E-07	0.07	8.36E-06	0.29	1.27E-04	1.13	3.95E-03	4.59	1.42E-06	0.05	1.67E-05	0.22	2	CHCl3 TTHM
326.2146@7.4439	2.72E-06	0.06	2.95E-05	0.23	2.11E-04	0.93	5.96E-04	4.52	4.31E-06	0.04	4.41E-05	0.18	2	CHCl3 TTHM
423.3225@8.147311	1.57E-06	0.06	8.31E-06	0.25	1.07E-04	1	2.23E-03	4.26	2.16E-06	0.05	1.56E-05	0.19	2	CHCl3 TTHM
477.2325@6.8104396	2.31E-06	-0.04	1.44E-05	-0.18	4.54E-05	-0.74	8.06E-05	-3.78	2.98E-06	-0.03	1.72E-05	-0.14	2	CHCl3 TTHM
232.9758@1.3577015	1.49E-06	0.06	9.84E-06	0.25	4.06E-05	1.04	1.15E-04	5.08	1.97E-06	0.05	1.27E-05	0.19	2	CHCl3 TTHM
530.1625@7.509525	5.85E-06	0.04	1.45E-05	0.16	7.04E-05	0.68	2.96E-04	3.28	6.36E-06	0.03	1.99E-05	0.13	2	CHCl3 TTHM
428.2174@5.9309206	5.85E-06	-0.02	2.47E-05	-0.07	7.01E-05	-0.3	2.10E-04	-1.45	6.99E-06	-0.01	2.95E-05	-0.05	2	CHCl3 TTHM
332.1633@7.3264585	6.55E-06	0.06	9.45E-06	0.28	3.91E-05	1.19	2.04E-04	5.71	6.22E-06	0.05	1.25E-05	0.22	2	CHCl3 TTHM
761.5589@9.035778	6.28E-06	0.02	1.33E-05	0.07	4.80E-05	0.31	2.91E-04	1.49	6.48E-06	0.01	1.73E-05	0.06	2	CHCl3 TTHM
537.281@6.9403844	9.81E-07	0.06	2.85E-05	0.23	5.85E-04	0.86	1.71E-02	3.2	2.20E-06	0.04	6.30E-05	0.17	2	CHCl3 TTHM
397.3185@6.13705	4.52E-05	0.04	7.65E-06	0.21	2.03E-05	0.91	1.66E-04	4.31	2.74E-05	0.04	9.29E-06	0.17	1	BDCM
420.7758@7.316014	1.67E-05	0.05	7.00E-06	0.24	2.29E-05	1.04	1.47E-04	4.96	1.21E-05	0.04	8.82E-06	0.19	1	BDCM
223.0168@3.196536	2.59E-05	-0.03	6.97E-06	-0.14	1.47E-05	-0.59	8.41E-05	-2.83	1.68E-05	-0.02	7.84E-06	-0.11	1	BDCM
322.2484@7.6077714	2.06E-05	0.06	7.47E-06	0.26	1.62E-05	1.14	1.94E-05	5.88	1.40E-05	0.05	8.02E-06	0.21	1	BDCM
253.0521@3.1947212	2.35E-05	-0.03	6.70E-06	-0.13	1.62E-05	-0.58	1.39E-04	-2.7	1.56E-05	-0.02	7.94E-06	-0.11	1	BDCM
301.225@4.7156005	6.71E-05	-0.03	1.40E-05	-0.15	8.29E-06	-0.7	3.81E-06	-3.77	3.93E-05	-0.03	1.11E-05	-0.12	1	CHBr3
550.3238@7.278686	2.81E-05	-0.03	1.07E-05	-0.14	8.25E-06	-0.62	2.99E-06	-3.36	1.84E-05	-0.02	8.86E-06	-0.11	1	CHBr3
277.1255@7.040886	1.04E-05	-0.04	1.51E-05	-0.16	1.81E-05	-0.72	6.71E-06	-3.9	9.15E-06	-0.03	1.37E-05	-0.13	1	CHBr3
256.0189@1.3662686	1.83E-05	0.06	1.70E-05	0.28	1.50E-05	1.24	5.62E-06	6.7	1.45E-05	0.05	1.47E-05	0.22	1	CHBr3
525.288@6.927325	6.29E-06	0.06	2.68E-05	0.24	1.74E-04	0.96	2.30E-03	4.18	8.06E-06	0.04	4.19E-05	0.18	1	CHCl3

153.0779@1.9292874	7.46E-06	-0.05	2.14E-05	-0.2	6.77E-05	-0.84	2.48E-04	-4.08	8.23E-06	-0.04	2.65E-05	-0.16	1	CHCl3
523.7939@7.0406313	8.26E-06	-0.04	9.00E-06	-0.16	1.46E-05	-0.69	2.40E-05	-3.5	6.95E-06	-0.03	9.20E-06	-0.12	1	TTHM
